# Supplementary material for: Transcriptomic changes associated with infection of Nicotiana benthamiana plants with tomato ringspot virus (genus Nepovirus) during the acute symptomatic stage and after symptom recovery
Source: PLoS One. 2025 Sep 2;20(9):e0328517. doi: 10.1371/journal.pone.0328517 (PMC12404439; doi:10.1371/journal.pone.0328517)
Supplement: S1 Fig — (PPTX) [file pone.0328517.s001.pptx]

## Slide 1
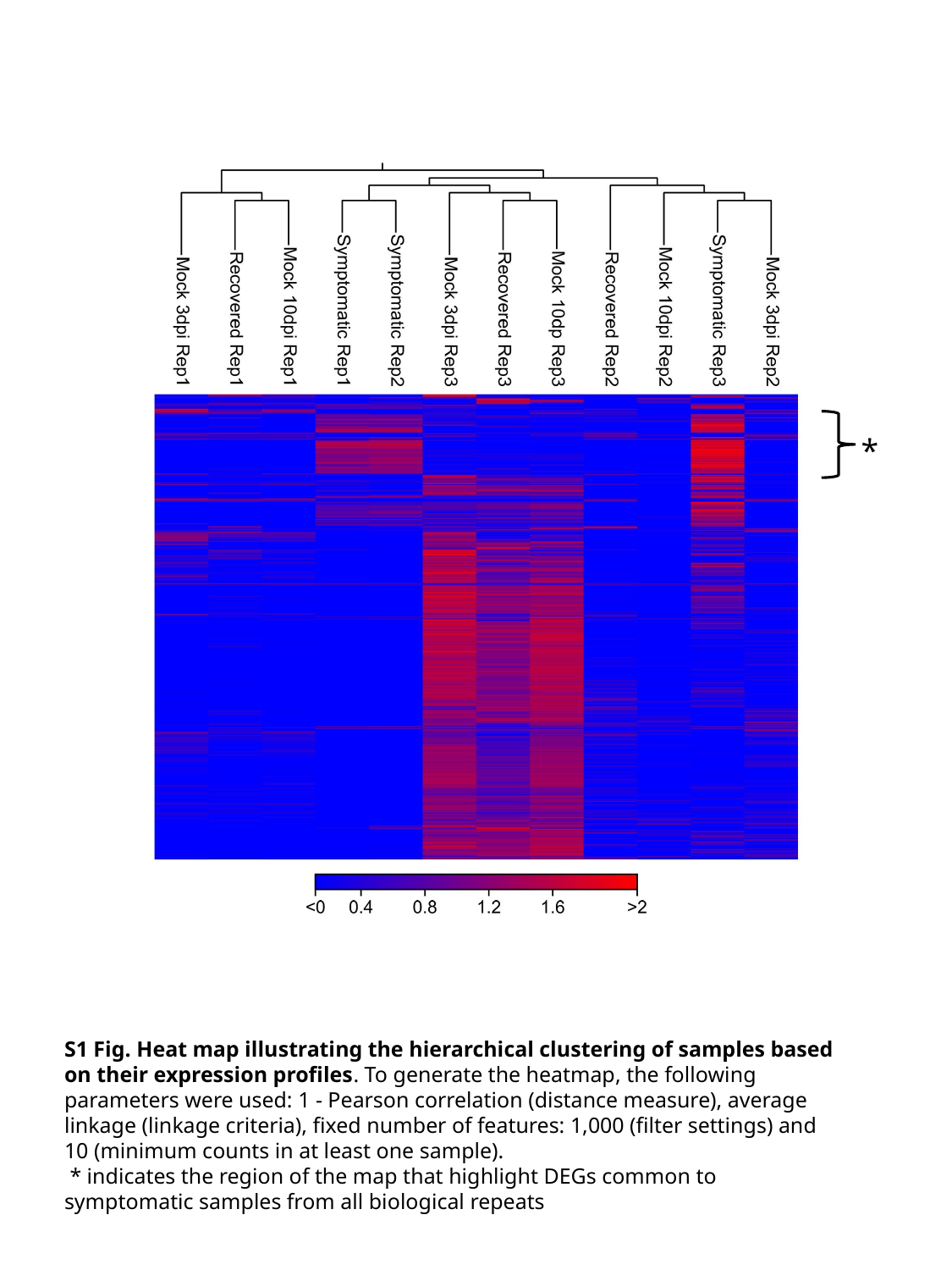

*
S1 Fig. Heat map illustrating the hierarchical clustering of samples based on their expression profiles. To generate the heatmap, the following parameters were used: 1 - Pearson correlation (distance measure), average linkage (linkage criteria), fixed number of features: 1,000 (filter settings) and 10 (minimum counts in at least one sample).
 * indicates the region of the map that highlight DEGs common to symptomatic samples from all biological repeats
