# Supplementary material for: Transcriptomic changes associated with infection of Nicotiana benthamiana plants with tomato ringspot virus (genus Nepovirus) during the acute symptomatic stage and after symptom recovery
Source: PLoS One. 2025 Sep 2;20(9):e0328517. doi: 10.1371/journal.pone.0328517 (PMC12404439; doi:10.1371/journal.pone.0328517)
Supplement: S2 Fig — (PPTX) [file pone.0328517.s002.pptx]

## Slide 1
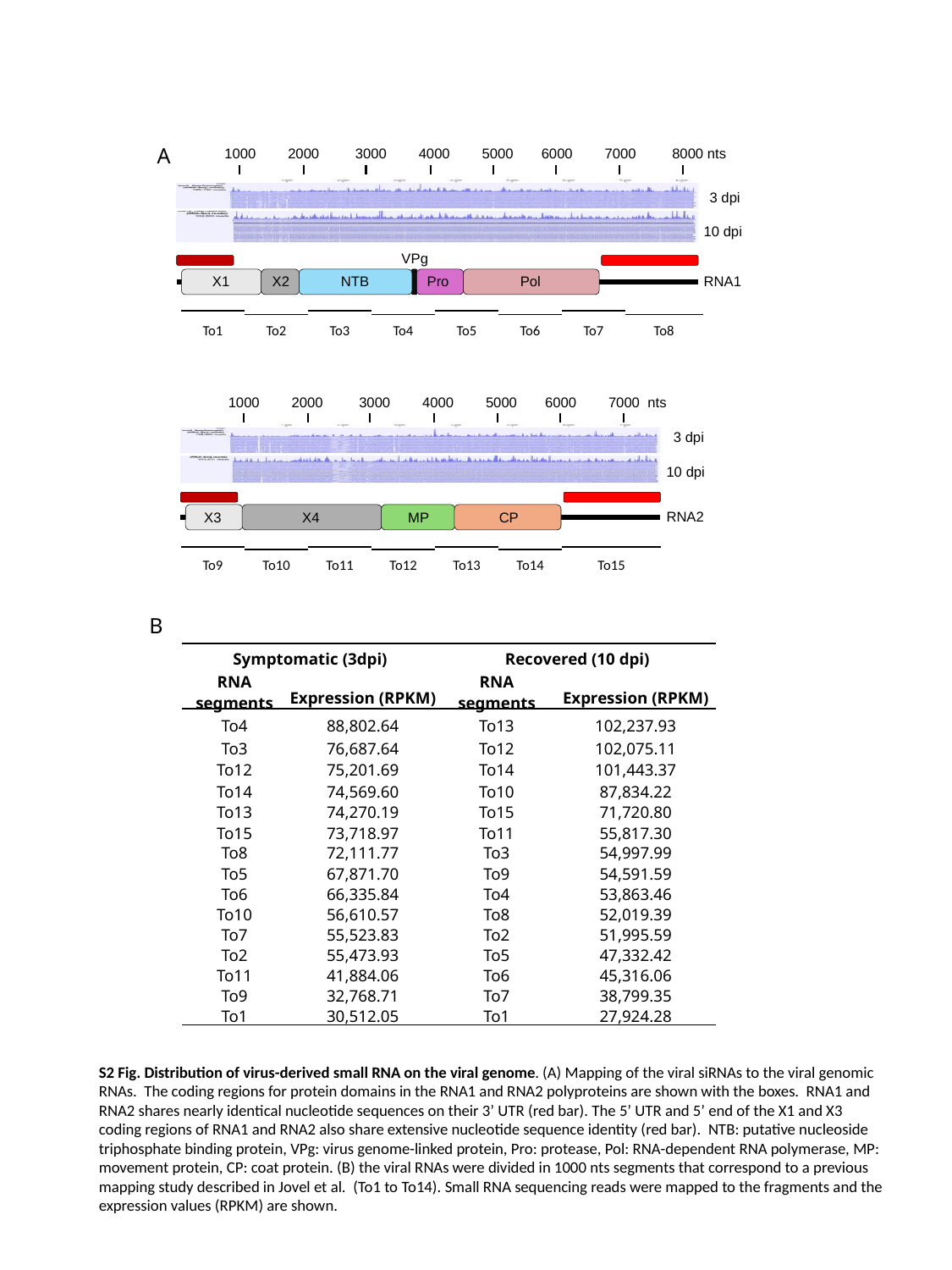

A
1000 2000 3000 4000 5000 6000 7000 8000 nts
3 dpi
10 dpi
VPg
RNA1
X1
X2
NTB
Pro
Pol
To1
To2
To3
To4
To5
To6
To7
To8
1000 2000 3000 4000 5000 6000 7000 nts
3 dpi
10 dpi
RNA2
X3
X4
MP
CP
To9
To10
To11
To12
To13
To14
To15
B
| Symptomatic (3dpi) | | Recovered (10 dpi) | |
| --- | --- | --- | --- |
| RNA segments | Expression (RPKM) | RNA segments | Expression (RPKM) |
| To4 | 88,802.64 | To13 | 102,237.93 |
| To3 | 76,687.64 | To12 | 102,075.11 |
| To12 | 75,201.69 | To14 | 101,443.37 |
| To14 | 74,569.60 | To10 | 87,834.22 |
| To13 | 74,270.19 | To15 | 71,720.80 |
| To15 | 73,718.97 | To11 | 55,817.30 |
| To8 | 72,111.77 | To3 | 54,997.99 |
| To5 | 67,871.70 | To9 | 54,591.59 |
| To6 | 66,335.84 | To4 | 53,863.46 |
| To10 | 56,610.57 | To8 | 52,019.39 |
| To7 | 55,523.83 | To2 | 51,995.59 |
| To2 | 55,473.93 | To5 | 47,332.42 |
| To11 | 41,884.06 | To6 | 45,316.06 |
| To9 | 32,768.71 | To7 | 38,799.35 |
| To1 | 30,512.05 | To1 | 27,924.28 |
S2 Fig. Distribution of virus-derived small RNA on the viral genome. (A) Mapping of the viral siRNAs to the viral genomic RNAs. The coding regions for protein domains in the RNA1 and RNA2 polyproteins are shown with the boxes. RNA1 and RNA2 shares nearly identical nucleotide sequences on their 3’ UTR (red bar). The 5’ UTR and 5’ end of the X1 and X3 coding regions of RNA1 and RNA2 also share extensive nucleotide sequence identity (red bar). NTB: putative nucleoside triphosphate binding protein, VPg: virus genome-linked protein, Pro: protease, Pol: RNA-dependent RNA polymerase, MP: movement protein, CP: coat protein. (B) the viral RNAs were divided in 1000 nts segments that correspond to a previous mapping study described in Jovel et al. (To1 to To14). Small RNA sequencing reads were mapped to the fragments and the expression values (RPKM) are shown.
