# Supplementary material for: Transcriptomic changes associated with infection of Nicotiana benthamiana plants with tomato ringspot virus (genus Nepovirus) during the acute symptomatic stage and after symptom recovery
Source: PLoS One. 2025 Sep 2;20(9):e0328517. doi: 10.1371/journal.pone.0328517 (PMC12404439; doi:10.1371/journal.pone.0328517)
Supplement: S3 Fig — (PPTX) [file pone.0328517.s003.pptx]

## Slide 1
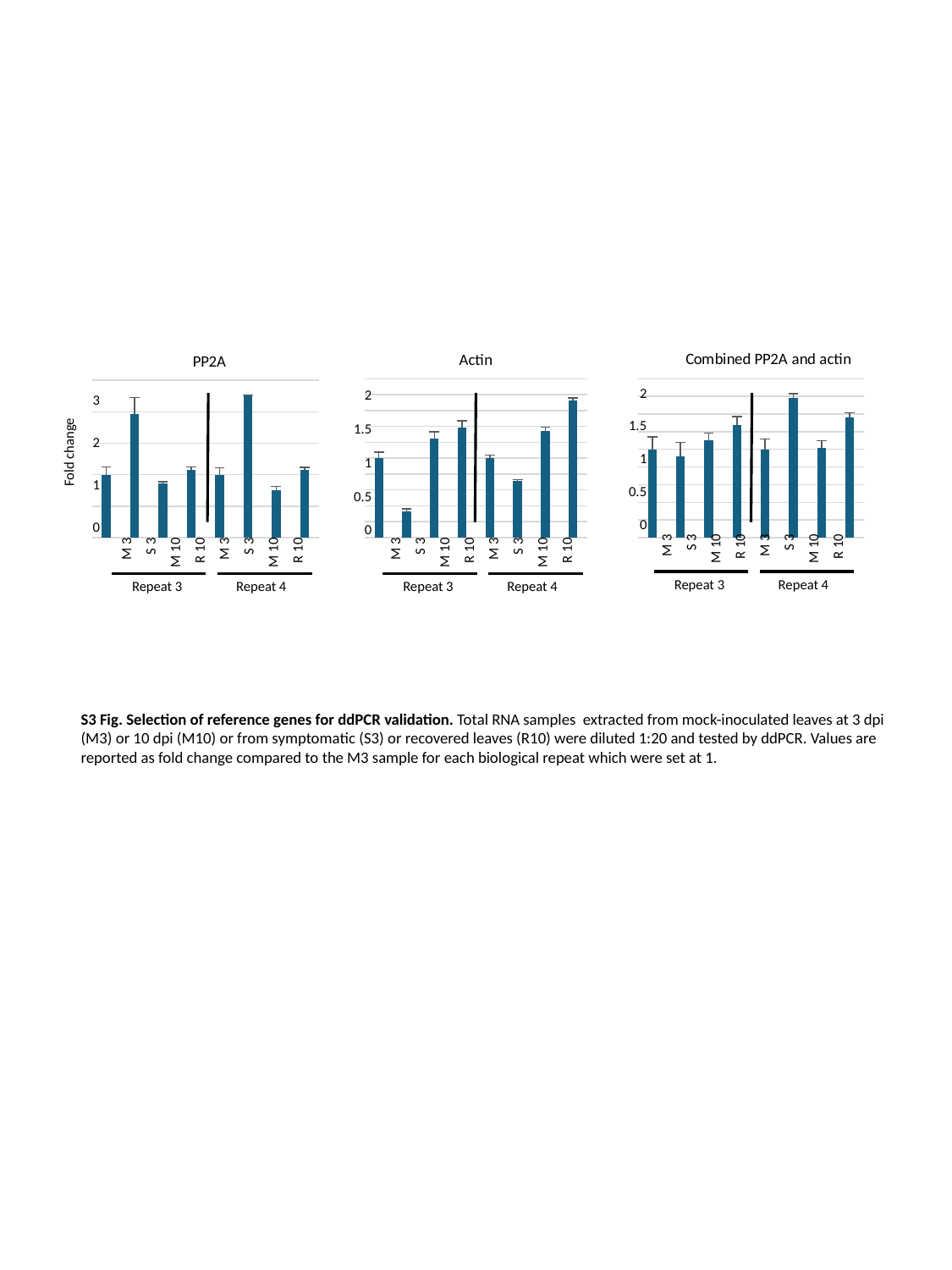

### Chart: Actin
| Category | |
|---|---|
| MK3 | 1.0 |
| Inoc3 | 0.32917038358608386 |
| MK10 | 1.247992863514719 |
| Rec10 | 1.3880463871543265 |
| R5_MK3 | 1.0 |
| R5_R3 | 0.7104913678618858 |
| R5_MK10 | 1.345285524568393 |
| R5_Rasp10 | 1.7304116865869854 |
### Chart: Combined PP2A and actin
| Category | |
|---|---|
| MK3 | 1.0 |
| Inoc3 | 0.9233946008435432 |
| MK10 | 1.1058015889759647 |
| Rec10 | 1.2736961357902086 |
| R5_MK3 | 1.0 |
| R5_R3 | 1.5828347363369546 |
| R5_MK10 | 1.0141828926400511 |
| R5_Rasp10 | 1.364956513958994 |
### Chart: PP2A
| Category | |
|---|---|
| MK3 | 1.0 |
| Inoc3 | 1.964677945978035 |
| MK10 | 0.8566340160284951 |
| Rec10 | 1.0733155238943306 |
| R5_MK3 | 1.0 |
| R5_R3 | 2.264080346593147 |
| R5_MK10 | 0.7556124458448208 |
| R5_Rasp10 | 1.0795588814493895 |3
2
1
0
2
1.5
1
0.5
0
2
1.5
1
0.5
0
Fold change
M 3
S 3
M 10
R 10
M 3
S 3
M 10
R 10
M 3
S 3
M 10
R 10
M 3
S 3
M 10
R 10
M 3
S 3
M 10
R 10
M 3
S 3
M 10
R 10
Repeat 3 Repeat 4
Repeat 3 Repeat 4
Repeat 3 Repeat 4
S3 Fig. Selection of reference genes for ddPCR validation. Total RNA samples extracted from mock-inoculated leaves at 3 dpi (M3) or 10 dpi (M10) or from symptomatic (S3) or recovered leaves (R10) were diluted 1:20 and tested by ddPCR. Values are reported as fold change compared to the M3 sample for each biological repeat which were set at 1.
