# Supplementary material for: Transcriptomic changes associated with infection of Nicotiana benthamiana plants with tomato ringspot virus (genus Nepovirus) during the acute symptomatic stage and after symptom recovery
Source: PLoS One. 2025 Sep 2;20(9):e0328517. doi: 10.1371/journal.pone.0328517 (PMC12404439; doi:10.1371/journal.pone.0328517)
Supplement: S1 Text — (DOCX) [file pone.0328517.s004.docx]

**S1 Text. Alignment of the nucleotide sequence of RNA1 comparing the original 2014 sequence to that assembled from RNA seq data for each biological repeat (Rep1, Rep2, Rep3).** Differences in the nucleotide sequences are highlighted with red font.

Rasp1-R1_2014 1 UUGCGAAACUUUCGGUGAUAUUCCGAAUUCUCUUUUCUCACUUUUAAUUUGUGCUGCUUUGAUUUCUUUUCGCAAUGUUUUCAGAUUGUCAUGGUGGUGG
Rasp1-R1_Rep1 1 UUGCGAAACUUUCGGUGAUAUUCCGAAUUCUCUUUUCUCACUUUUAAUUUGUGCUGCUUUGAUUUCUUUUCGCAAUGUUUUCAGAUUGUCAUGGUGGUGG
Rasp1-R1_Rep2 1 UUGCGAAACUUUCGGUGAUAUUCCGAAUUCUCUUUUCUCACUUUUAAUUUGUGCUGCUUUGAUUUCUUUUCGCAAUGUUUUCAGAUUGUCAUGGUGGUGG
Rasp1-R1_Rep3 1 NUGCGAAACUUUCGGUGAUAUUCCGAAUUCUCUUUUCUCACUUUUAAUUUGUGCUGCUUUGAUUUCUUUUCGCAAUGUUUUCAGAUUGUCAUGGUGGUGG

Rasp1-R1_2014 101 CAACCAUGCUAGGUUGCCAUCAAAGGCUGCUUUCCGUCGGGCUAUGUCCGACGGAGAUCUUGAUCGCGAGGGUCGCUUCCCUUGCGGGUGUCUUGCACAG
Rasp1-R1_Rep1 101 CAACCAUGCUAGGUUGCCAUCAAAGGCUGCUUUCCGUCGGGCUAUGUCCGACGGAGAUCUUGAUCGCGAGGGUCGCUUCCCUUGCGGGUGUCUUGCACAG
Rasp1-R1_Rep2 101 CAACCAUGCUAGGUUGCCAUCAAAGGCUGCUUUCCGUCGGGCUAUGUCCGACGGAGAUCUUGAUCGCGAGGGUCGCUUCCCUUGCGGGUGUCUUGCACAG
Rasp1-R1_Rep3 101 CAACCAUGCUAGGUUGCCAUCAAAGGCUGCUUUCCGUCGGGCUAUGUCCGACGGAGAUCUUGAUCGCGAGGGUCGCUUCCCUUGCGGGUGUCUUGCACAG

Rasp1-R1_2014 201 UUUGAUGUGCAAAUCCCCUCUCCUGCAAAGGCACAGGAGAAAGUCGUAGGUAGGUCCGCUGACCUCCAGGGUAAUGUUGGUCCCCUCAAGAAGCAACGCU
Rasp1-R1_Rep1 201 UUUGAUGUGCAAAUCCCCUCUCCUGCAAAGGCACAGGAGAAAGUCGUAGGUAGGUCCGCUGACCUCCAGGGUAAUGUUGGUCCCCUCAAGAAGCAACGCU
Rasp1-R1_Rep2 201 UUUGAUGUGCAAAUCCCCUCUCCUGCAAAGGCACAGGAGAAAGUCGUAGGUAGGUCCGCUGACCUCCAGGGUAAUGUUGGUCCCCUCAAGAAGCAACGCU
Rasp1-R1_Rep3 201 UUUGAUGUGCAAAUCCCCUCUCCUGCAAAGGCACAGGAGAAAGUCGUAGGUAGGUCCGCUGACCUCCAGGGUAAUGUUGGUCCCCUCAAGAAGCAACGCU

Rasp1-R1_2014 301 GCGAUGUUGUGGUCGCAGUUUUUGGACCUCCUCCGUUGGAGUUGGUUUACCCGGCCCGGGUAGGGCAACAUAGGUUGGACCAACCUUCAAAAGGUCCCUU
Rasp1-R1_Rep1 301 GCGAUGUUGUGGUCGCAGUUUUUGGACCUCCUCCGUUGGAGUUGGUUUACCCGGCCCGGGUAGGGCAACAUAGGUUGGACCAACCUUCAAAAGGUCCCUU
Rasp1-R1_Rep2 301 GCGAUGUUGUGGUCGCAGUUUUUGGACCUCCUCCGUUGGAGUUGGUUUACCCGGCCCGGGUAGGGCAACAUAGGUUGGACCAACCUUCAAAAGGUCCCUU
Rasp1-R1_Rep3 301 GCGAUGUUGUGGUCGCAGUUUUUGGACCUCCUCCGUUGGAGUUGGUUUACCCGGCCCGGGUAGGGCAACAUAGGUUGGACCAACCUUCAAAAGGUCCCUU

Rasp1-R1_2014 401 GGCAGUUCCUGCUGCCAAGCAAACCUCCACUGCAAUGGAGGUUGUUCUUUCUGUUAGGGAGGCAGCUUUCACCGCCCCCUGGCUCCUUCGCUCCUGCAAG
Rasp1-R1_Rep1 401 GGCAGUUCCUGCUGCCAAGCAAACCUCCACUGCAAUGGAGGUUGUUCUUUCUGUUAGGGAGGCAGCUUUCACCGCCCCCUGGCUCCUUCGCUCCUGCAAG
Rasp1-R1_Rep2 401 GGCAGUUCCUGCUGCCAAGCAAACCUCCACUGCAAUGGAGGUUGUUCUUUCUGUUAGGGAGGCAGCUUUCACCGCCCCCUGGCUCCUUCGCUCCUGCAAG
Rasp1-R1_Rep3 401 GGCAGUUCCUGCUGCCAAGCAAACCUCCACUGCAAUGGAGGUUGUUCUUUCUGUUAGGGAGGCAGCUUUCACCGCCCCCUGGCUCCUUCGCUCCUGCAAG

Rasp1-R1_2014 501 AGCGGAGUUCCCCCCCCCCCCCCCCCCAUGACACAAAGGCAGCAGUUUGCUGCCUUGAAGAGGAGGCUGGUCCAAAAGGGCCAGCAAACUAUUCGCGAGC
Rasp1-R1_Rep1 501 AGCGGAGUUCCCCCCCCCCCCCCCCCCAUGACACAAAGGCAGCAGUUUGCUGCCUUGAAGAGGAGGCUGGUCCAAAAGGGCCAGCAAACUAUUCGCGAGC
Rasp1-R1_Rep2 501 AGCGGAGUUCCCCCCCCCCCCCCCCCCAUGACACAAAGGCAGCAGUUUGCUGCCUUGAAGAGGAGGCUGGUCCAAAAGGGCCAGCAAACUAUUCGCGAGC
Rasp1-R1_Rep3 501 AGCGGAGUUCCCCCCCCCCCCCCCCCCAUGACACAAAGGCAGCAGUUUGCUGCCUUGAAGAGGAGGCUGGUCCAAAAGGGCCAGCAAACUAUUCGCGAGC

Rasp1-R1_2014 601 UCAUCCGAGCUCGCAAGGCGGCUAAGUAUGCCGCCAUUGCCGCCCAGAAAAGGGCGGCUGCUGUGGCUGCCCAAAAGGCAGCAGCUGGGGCCCCGCGCCU
Rasp1-R1_Rep1 601 UCAUCCGAGCUCGCAAGGCGGCUAAGUAUGCCGCCAUUGCCGCCCAGAAAAGGGCGGCUGCUGUGGCUGCCCAAAAGGCAGCAGCUGGGGCCCCGCGCCU
Rasp1-R1_Rep2 601 UCAUCCGAGCUCGCAAGGCGGCUAAGUAUGCCGCCAUUGCCGCCCAGAAAAGGGCGGCUGCUGUGGCUGCCCAAAAGGCAGCAGCUGGGGCCCCGCGCCU
Rasp1-R1_Rep3 601 UCAUCCGAGCUCGCAAGGCGGCUAAGUAUGCCGCCAUUGCCGCCCAGAAAAGGGCGGCUGCUGUGGCUGCCCAAAAGGCAGCAGCUGGGGCCCCGCGCCU

Rasp1-R1_2014 701 CGCGGCCCAAAGGGCCGCAGUUGCUAAGAUCCUUCGGGAUCGGCAACUGGCUUCUCUUCCCCCUCCUCCUCCUCCUUCUGCUGCCAGAUUGGCAGCUGAG
Rasp1-R1_Rep1 701 CGCGGCCCAAAGGGCCGCAGUUGCUAAGAUCCUUCGGGAUCGGCAACUGGCUUCUCUUCCCCCUCCUCCUCCUCCUUCUGCUGCCAGAUUGGCAGCUGAG
Rasp1-R1_Rep2 701 CGCGGCCCAAAGGGCCGCAGUUGCUAAGAUCCUUCGGGAUCGGCAACUGGCUUCUCUUCCCCCUCCUCCUCCUCCUUCUGCUGCCAGAUUGGCAGCUGAG
Rasp1-R1_Rep3 701 CGCGGCCCAAAGGGCCGCAGUUGCUAAGAUCCUUCGGGAUCGGCAACUGGCUUCUCUUCCCCCUCCUCCUCCUCCUUCUGCUGCCAGAUUGGCAGCUGAG

Rasp1-R1_2014 801 GCCGAAUUGGCCUCUAAAGCAGCCUCUCUUUUGAGGCUAAAGGCCUUUAAAAAGGCCAGCAGGGUUCGCCCUGCUUUAAAUAUUUUUUUUCCUCUCCCCC
Rasp1-R1_Rep1 801 GCCGAAUUGGCCUCUAAAGCAGCCUCUCUUUUGAGGCUAAAGGCCUUUAAAAAGGCCAGCAGGGUUCGCCCUGCUUUAAAUAUUUUUUUUCCUCUCCCCC
Rasp1-R1_Rep2 801 GCCGAAUUGGCCUCUAAAGCAGCCUCUCUUUUGAGGCUAAAGGCCUUUAAAAAGGCCAGCAGGGUUCGCCCUGCUUUAAAUAUUUUUUUUCCUCUCCCCC
Rasp1-R1_Rep3 801 GCCGAAUUGGCCUCUAAAGCAGCCUCUCUUUUGAGGCUAAAGGCCUUUAAAAAGGCCAGCAGGGUUCGCCCUGCUUUAAAUAUUUUUUUUCCUCUCCCCC

Rasp1-R1_2014 901 CCCCUGAGCGGGUUCGCGAUCCCGCCCUUCUUGAGCGUCUGAGGGUGGCAACACCCUCACGCGCCCGUGUCGCUUCCAAGCGACAAAGAGAGUUCUCCCC
Rasp1-R1_Rep1 901 CCCCUGAGCGGGUUCGCGAUCCCGCCCUUCUUGAGCGUCUGAGGGUGGCAACACCCUCACGCGCCCGUGUCGCUUCCAAGCGACAAAGAGAGUUCUCCCC
Rasp1-R1_Rep2 901 CCCCUGAGCGGGUUCGCGAUCCCGCCCUUCUUGAGCGUCUGAGGGUGGCAACACCCUCACGCGCCCGUGUCGCUUCCAAGCGACAAAGAGAGUUCUCCCC
Rasp1-R1_Rep3 901 CCCCUGAGCGGGUUCGCGAUCCCGCCCUUCUUGAGCGUCUGAGGGUGGCAACACCCUCACGCGCCCGUGUCGCUUCCAAGCGACAAAGAGAGUUCUCCCC

Rasp1-R1_2014 1001 UCCCCCCCUCGCCACCCAAGUUAUGGUGGCUGCUUGCGCUUCUCAUCAGGAAGCCUAUGACAAGUGUCGCUCCCUUUUGAUUUCGGAGUGGCCUGAGAGC
Rasp1-R1_Rep1 1001 UCCCCCCCUCGCCACCCAAGUUAUGGUGGCUGCUUGCGCUUCUCAUCAGGAAGCCUAUGACAAGUGUCGCUCCCUUUUGAUUUCGGAGUGGCCUGAGAGC
Rasp1-R1_Rep2 1001 UCCCCCCCUCGCCACCCAAGUUAUGGUGGCUGCUUGCGCUUCUCAUCAGGAAGCCUAUGACAAGUGUCGCUCCCUUUUGAUUUCGGAGUGGCCUGAGAGC
Rasp1-R1_Rep3 1001 UCCCCCCCUCGCCACCCAAGUUAUGGUGGCUGCUUGCGCUUCUCAUCAGGAAGCCUAUGACAAGUGUCGCUCCCUUUUGAUUUCGGAGUGGCCUGAGAGC

Rasp1-R1_2014 1101 GAGCAACUUUUUGGACCGCUUGUUUUUGCUGAGGGUUGGAAAUAUGUUCCCGGUAUGUUAUUACAAUAUCGCUUGUGCAUUGUAUUUUGCAUGGUGAGGG
Rasp1-R1_Rep1 1101 GAGCAACUUUUUGGACCGCUUGUUUUUGCUGAGGGUUGGAAAUAUGUUCCCGGUAUGUUAUUACAAUAUCGCUUGUGCAUUGUAUUUUGCAUGGUGAGGG
Rasp1-R1_Rep2 1101 GAGCAACUUUUUGGACCGCUUGUUUUUGCUGAGGGUUGGAAAUAUGUUCCCGGUAUGUUAUUACAAUAUCGCUUGUGCAUUGUAUUUUGCAUGGUGAGGG
Rasp1-R1_Rep3 1101 GAGCAACUUUUUGGACCGCUUGUUUUUGCUGAGGGUUGGAAAUAUGUUCCCGGUAUGUUAUUACAAUAUCGCUUGUGCAUUGUAUUUUGCAUGGUGAGGG

Rasp1-R1_2014 1201 ACGUAAUGCCAGCCUUAUCUAUUGUUGCAGACACUCUCCAUGCUCUUCGAAGUGGCGUAGCACCUUGCAUUGUUUUUAAAAAUGCCAUGGUCACUGCCAA
Rasp1-R1_Rep1 1201 ACGUAAUGCCAGCCUUAUCUAUUGUUGCAGACACUCUCCAUGCUCUUCGAAGUGGCGUAGCACCUUGCAUUGUUUUUAAAAAUGCCAUGGUCACUGCCAA
Rasp1-R1_Rep2 1201 ACGUAAUGCCAGCCUUAUCUAUUGUUGCAGACACUCUCCAUGCUCUUCGAAGUGGCGUAGCACCUUGCAUUGUUUUUAAAAAUGCCAUGGUCACUGCCAA
Rasp1-R1_Rep3 1201 ACGUAAUGCCAGCCUUAUCUAUUGUUGCAGACACUCUCCAUGCUCUUCGAAGUGGCGUAGCACCUUGCAUUGUUUUUAAAAAUGCCAUGGUCACUGCCAA

Rasp1-R1_2014 1301 UCAGAUAUUGGAGUUGUCUCACUCUUCCCACGCAGCCCAAGGCCUCGGCGCCUUUUUAAGUCGAGGUAAACAGGCUGCCCUUGAUUUUGCUAGUGGUGCG
Rasp1-R1_Rep1 1301 UCAGAUAUUGGAGUUGUCUCACUCUUCCCACGCAGCCCAAGGCCUCGGCGCCUUUUUAAGUCGAGGUAAACAGGCUGCCCUUGAUUUUGCUAGUGGUGCG
Rasp1-R1_Rep2 1301 UCAGAUAUUGGAGUUGUCUCACUCUUCCCACGCAGCCCAAGGCCUCGGCGCCUUUUUAAGUCGAGGUAAACAGGCUGCCCUUGAUUUUGCUAGUGGUGCG
Rasp1-R1_Rep3 1301 UCAGAUAUUGGAGUUGUCUCACUCUUCCCACGCAGCCCAAGGCCUCGGCGCCUUUUUAAGUCGAGGUAAACAGGCUGCCCUUGAUUUUGCUAGUGGUGCG

Rasp1-R1_2014 1401 UACAAUUGUGCUGCCGCUAAGAUCGUGAAAGGUGCCACAGCCGUCGUUGAUAAAGCUUCGGAGGUGGUAGUAGAUAAACUUUUUGUCCCAUUUGUAAAUU
Rasp1-R1_Rep1 1401 UACAAUUGUGCUGCCGCUAAGAUCGUGAAAGGUGCCACAGCCGUCGUUGAUAAAGCUUCGGAGGUGGUAGUAGAUAAACUUUUUGUCCCAUUUGUAAAUU
Rasp1-R1_Rep2 1401 UACAAUUGUGCUGCCGCUAAGAUCGUGAAAGGUGCCACAGCCGUCGUUGAUAAAGCUUCGGAGGUGGUAGUAGAUAAACUUUUUGUCCCAUUUGUAAAUU
Rasp1-R1_Rep3 1401 UACAAUUGUGCUGCCGCUAAGAUCGUGAAAGGUGCCACAGCCGUCGUUGAUAAAGCUUCGGAGGUGGUAGUAGAUAAACUUUUUGUCCCAUUUGUAAAUU

Rasp1-R1_2014 1501 UGUUGCGGGGACAUUUUGAUGAUACCAUAGGUAGAUGGAUCCCUAAACUACUAGGUGCCGCAGACAAAAUUGAAAAUUUGUGGCGGUGGUCAGUUCAAUG
Rasp1-R1_Rep1 1501 UGUUGCGGGGACAUUUUGAUGAUACCAUAGGUAGAUGGAUCCCUAAACUACUAGGUGCCGCAGACAAAAUUGAAAAUUUGUGGCGGUGGUCAGUUCAAUG
Rasp1-R1_Rep2 1501 UGUUGCGGGGACAUUUUGAUGAUACCAUAGGUAGAUGGAUCCCUAAACUACUAGGUGCCGCAGACAAAAUUGAAAAUUUGUGGCGGUGGUCAGUUCAAUG
Rasp1-R1_Rep3 1501 UGUUGCGGGGACAUUUUGAUGAUACCAUAGGUAGAUGGAUCCCUAAACUACUAGGUGCCGCAGACAAAAUUGAAAAUUUGUGGCGGUGGUCAGUUCAAUG

Rasp1-R1_2014 1601 GGCCCAAAACAUGACUAACAAAUUAGAUUUGUCUUUGCGCGUUUUGCGCGGAUCAGCCCUACUUGGGGUUGGUCUUUUGUUAGUUUCUGGUAUUUUGUAU
Rasp1-R1_Rep1 1601 GGCCCAAAACAUGACUAACAAAUUAGAUUUGUCUUUGCGCGUUUUGCGCGGAUCAGCCCUACUUGGGGUUGGUCUUUUGUUAGUUUCUGGUAUUUUGUAU
Rasp1-R1_Rep2 1601 GGCCCAAAACAUGACUAACAAAUUAGAUUUGUCUUUGCGCGUUUUGCGCGGAUCAGCCCUACUUGGGGUUGGUCUUUUGUUAGUUUCUGGUAUUUUGUAU
Rasp1-R1_Rep3 1601 GGCCCAAAACAUGACUAACAAAUUAGAUUUGUCUUUGCGCGUUUUGCGCGGAUCAGCCCUACUUGGGGUUGGUCUUUUGUUAGUUUCUGGUAUUUUGUAU

Rasp1-R1_2014 1701 UUUGCGGAGCAAUUGCUCCGCUCUUUUGGUUUAGUUAUCGUUGCAGGCUCUUGUAUUUCCAUGUUUGUGGGAGGCUUGUUGCUUGCAUAUUCUGGUAGCC
Rasp1-R1_Rep1 1701 UUUGCGGAGCAAUUGCUCCGCUCUUUUGGUUUAGUUAUCGUUGCAGGCUCUUGUAUUUCCAUGUUUGUGGGAGGCUUGUUGCUUGCAUAUUCUGGUAGCC
Rasp1-R1_Rep2 1701 UUUGCGGAGCAAUUGCUCCGCUCUUUUGGUUUAGUUAUCGUUGCAGGCUCUUGUAUUUCCAUGUUUGUGGGAGGCUUGUUGCUUGCAUAUUCUGGUAGCC
Rasp1-R1_Rep3 1701 UUUGCGGAGCAAUUGCUCCGCUCUUUUGGUUUAGUUAUCGUUGCAGGCUCUUGUAUUUCCAUGUUUGUGGGAGGCUUGUUGCUUGCAUAUUCUGGUAGCC

Rasp1-R1_2014 1801 UAACCGGUAUUUUUGAUGAGCAAAUGAUGCGGGUUCGUGGUAUUUUAUGCGAAAUUCCCAUGUUGCUCUAUUUGAAAGCGCAACCAGAUCCUCUUUUCCC
Rasp1-R1_Rep1 1801 UAACCGGUAUUUUUGAUGAGCAAAUGAUGCGGGUUCGUGGUAUUUUAUGCGAAAUUCCCAUGUUGCUCUAUUUGAAAGCGCAACCAGAUCCUCUUUUCCC
Rasp1-R1_Rep2 1801 UAACCGGUAUUUUUGAUGAGCAAAUGAUGCGGGUUCGUGGUAUUUUAUGCGAAAUUCCCAUGUUGCUCUAUUUGAAAGCGCAACCAGAUCCUCUUUUCCC
Rasp1-R1_Rep3 1801 UAACCGGUAUUUUUGAUGAGCAAAUGAUGCGGGUUCGUGGUAUUUUAUGCGAAAUUCCCAUGUUGCUCUAUUUGAAAGCGCAACCAGAUCCUCUUUUCCC

Rasp1-R1_2014 1901 GAAGCGCGCCGGUGGUGGGGCCUCCGUUCAGGGCCUCACGGAUGUUUUUGGAGUUCCCCUCAGCAUCAUGAAUGCUUUGGGAGAUGGAUUAGUCCAUCAC
Rasp1-R1_Rep1 1901 GAAGCGCGCCGGUGGUGGGGCCUCCGUUCAGGGCCUCACGGAUGUUUUUGGAGUUCCCCUCAGCAUCAUGAAUGCUUUGGGAGAUGGAUUAGUCCAUCAC
Rasp1-R1_Rep2 1901 GAAGCGCGCCGGUGGUGGGGCCUCCGUUCAGGGCCUCACGGAUGUUUUUGGAGUUCCCCUCAGCAUCAUGAAUGCUUUGGGAGAUGGAUUAGUCCAUCAC
Rasp1-R1_Rep3 1901 GAAGCGCGCCGGUGGUGGGGCCUCCGUUCAGGGCCUCACGGAUGUUUUUGGAGUUCCCCUCAGCAUCAUGAAUGCUUUGGGAGAUGGAUUAGUCCAUCAC

Rasp1-R1_2014 2001 UCUCUUGACACUUUGCAGUUGAUGGGGAAGUUUGGUGCAGCUAUGGAUAAUGUCCGUAAGGGCAUUACCUGCAUGAGGUCUUUUGUUUCGUGGCUGAUGG
Rasp1-R1_Rep1 2001 UCUCUUGACACUUUGCAGUUGAUGGGGAAGUUUGGUGCAGCUAUGGAUAAUGUCCGUAAGGGCAUUACCUGCAUGAGGUCUUUUGUUUCGUGGCUGAUGG
Rasp1-R1_Rep2 2001 UCUCUUGACACUUUGCAGUUGAUGGGGAAGUUUGGUGCAGCUAUGGAUAAUGUCCGUAAGGGCAUUACCUGCAUGAGGUCUUUUGUUUCGUGGCUGAUGG
Rasp1-R1_Rep3 2001 UCUCUUGACACUUUGCAGUUGAUGGGGAAGUUUGGUGCAGCUAUGGAUAAUGUCCGUAAGGGCAUUACCUGCAUGAGGUCUUUUGUUUCGUGGCUGAUGG

Rasp1-R1_2014 2101 AACAUUUGGCCCUAGCUCUUGAUAAAAUAACAGGCAAGCGUACCGCUUUUUUUAGGGAACUUGCCACAUUAAUUAAUUUUGAUGUUGAGAAGUGGGUCCG
Rasp1-R1_Rep1 2101 AACAUUUGGCCCUAGCUCUUGAUAAAAUAACAGGCAAGCGUACCGCUUUUUUUAGGGAACUUGCCACAUUAAUUAAUUUUGAUGUUGAGAAGUGGGUCCG
Rasp1-R1_Rep2 2101 AACAUUUGGCCCUAGCUCUUGAUAAAAUAACAGGCAAGCGUACCGCUUUUUUUAGGGAACUUGCCACAUUAAUUAAUUUUGAUGUUGAGAAGUGGGUCCG
Rasp1-R1_Rep3 2101 AACAUUUGGCCCUAGCUCUUGAUAAAAUAACAGGCAAGCGUACCGCUUUUUUUAGGGAACUUGCCACAUUAAUUAAUUUUGAUGUUGAGAAGUGGGUCCG

Rasp1-R1_2014 2201 AGAUUCUCAGCAGUAUUUAUUGGCUGCUGAAAUUUAUGUUGAUGGUGAUACUGUUGUCAUGGAUACGUGUCGCCAUUUACUUGAUAAGGGCCUGAAGCUC
Rasp1-R1_Rep1 2201 AGAUUCUCAGCAGUAUUUAUUGGCUGCUGAAAUUUAUGUUGAUGGUGAUACUGUUGUCAUGGAUACGUGUCGCCAUUUACUUGAUAAGGGCCUGAAGCUC
Rasp1-R1_Rep2 2201 AGAUUCUCAGCAGUAUUUAUUGGCUGCUGAAAUUUAUGUUGAUGGUGAUACUGUUGUCAUGGAUACGUGUCGCCAUUUACUUGAUAAGGGCCUGAAGCUC
Rasp1-R1_Rep3 2201 AGAUUCUCAGCAGUAUUUAUUGGCUGCUGAAAUUUAUGUUGAUGGUGAUACUGUUGUCAUGGAUACGUGUCGCCAUUUACUUGAUAAGGGCCUGAAGCUC

Rasp1-R1_2014 2301 CAACGAAUGAUGGUCAGUUCUAAGUCUGGUACUUCAUUCAAUUAUGGCCGUCUCGUUGGGGAUCUCGUUAAGAGGUUGAGCGAUCUGCAUAAACGCUAUU
Rasp1-R1_Rep1 2301 CAACGAAUGAUGGUCAGUUCUAAGUCUGGUACUUCAUUCAAUUAUGGCCGUCUCGUUGGGGAUCUCGUUAAGAGGUUGAGCGAUCUGCAUAAACGCUAUU
Rasp1-R1_Rep2 2301 CAACGAAUGAUGGUCAGUUCUAAGUCUGGUACUUCAUUCAAUUAUGGCCGUCUCGUUGGGGAUCUCGUUAAGAGGUUGAGCGAUCUGCAUAAACGCUAUU
Rasp1-R1_Rep3 2301 CAACGAAUGAUGGUCAGUUCUAAGUCUGGUACUUCAUUCAAUUAUGGCCGUCUCGUUGGGGAUCUCGUUAAGAGGUUGAGCGAUCUGCAUAAACGCUAUU

Rasp1-R1_2014 2401 GUGCUUCGGGACGCCGUGUGCAUUAUAGACUUGCGCCCUAUUGGGUGUAUUUGUACGGUGGCCCGAGGUGUGGUAAAUCCCUCUUUGCUCAGAGUUUCAU
Rasp1-R1_Rep1 2401 GUGCUUCGGGACGCCGUGUGCAUUAUAGACUUGCGCCCUAUUGGGUGUAUUUGUACGGUGGCCCGAGGUGUGGUAAAUCCCUCUUUGCUCAGAGUUUCAU
Rasp1-R1_Rep2 2401 GUGCUUCGGGACGCCGUGUGCAUUAUAGACUUGCGCCCUAUUGGGUGUAUUUGUACGGUGGCCCGAGGUGUGGUAAAUCCCUCUUUGCUCAGAGUUUCAU
Rasp1-R1_Rep3 2401 GUGCUUCGGGACGCCGUGUGCAUUAUAGACUUGCGCCCUAUUGGGUGUAUUUGUACGGUGGCCCGAGGUGUGGUAAAUCCCUCUUUGCUCAGAGUUUCAU

Rasp1-R1_2014 2501 GAAUACGGCGGUGGAUUUUAUGGGCACCACUACCGAUAAUUGCUAUUUCAAAAAUGCCCGUGAUGAUUUUUGGAGUGGAUAUCGACAAGAAGCAAUUUGC
Rasp1-R1_Rep1 2501 GAAUACGGCGGUGGAUUUUAUGGGCACCACUACCGAUAAUUGCUAUUUCAAAAAUGCCCGUGAUGAUUUUUGGAGUGGAUAUCGACAAGAAGCAAUUUGC
Rasp1-R1_Rep2 2501 GAAUACGGCGGUGGAUUUUAUGGGCACCACUACCGAUAAUUGCUAUUUCAAAAAUGCCCGUGAUGAUUUUUGGAGUGGAUAUCGACAAGAAGCAAUUUGC
Rasp1-R1_Rep3 2501 GAAUACGGCGGUGGAUUUUAUGGGCACCACUACCGAUAAUUGCUAUUUCAAAAAUGCCCGUGAUGAUUUUUGGAGUGGAUAUCGACAAGAAGCAAUUUGC

Rasp1-R1_2014 2601 UGUGUCGAUGAUCUUUCUUCUUGCGAGACGCAACCUUCUAUUGAGUCGGAAUUCAUUCAAUUGAUAACGACAAUGAGAUAUGGGUUGAAUAUGGCAGGAG
Rasp1-R1_Rep1 2601 UGUGUCGAUGAUCUUUCUUCUUGCGAGACGCAACCUUCUAUUGAGUCGGAAUUCAUUCAAUUGAUAACGACAAUGAGAUAUGGGUUGAAUAUGGCAGGAG
Rasp1-R1_Rep2 2601 UGUGUCGAUGAUCUUUCUUCUUGCGAGACGCAACCUUCUAUUGAGUCGGAAUUCAUUCAAUUGAUAACGACAAUGAGAUAUGGGUUGAAUAUGGCAGGAG
Rasp1-R1_Rep3 2601 UGUGUCGAUGAUCUUUCUUCUUGCGAGACGCAACCUUCUAUUGAGUCGGAAUUCAUUCAAUUGAUAACGACAAUGAGAUAUGGGUUGAAUAUGGCAGGAG

Rasp1-R1_2014 2701 UGGAAGAGAAGGGAGCUCAAUUUAAUUCUAAGAUGGUUAUUACGACCUCUAAUUUUUUCACUGCUCCCACCACUGCCAAGAUUGCUGACAAGGCUGCCUA
Rasp1-R1_Rep1 2701 UGGAAGAGAAGGGAGCUCAAUUUAAUUCUAAGAUGGUUAUUACGACCUCUAAUUUUUUCACUGCUCCCACCACUGCCAAGAUUGCUGACAAGGCUGCCUA
Rasp1-R1_Rep2 2701 UGGAAGAGAAGGGAGCUCAAUUUAAUUCUAAGAUGGUUAUUACGACCUCUAAUUUUUUCACUGCUCCCACCACUGCCAAGAUUGCUGACAAGGCUGCCUA
Rasp1-R1_Rep3 2701 UGGAAGAGAAGGGAGCUCAAUUUAAUUCUAAGAUGGUUAUUACGACCUCUAAUUUUUUCACUGCUCCCACCACUGCCAAGAUUGCUGACAAGGCUGCCUA

Rasp1-R1_2014 2801 UAAUCAUCGGAGACAUGCUUGUGUCCUUGUUCAGCGAAAGGAAGGGGUGAAGUAUGACCCCAGUAAUCCUGCUGCUGCCGCGGAGGCAAUGUUUGUUGAU
Rasp1-R1_Rep1 2801 UAAUCAUCGGAGACAUGCUUGUGUCCUUGUUCAGCGAAAGGAAGGGGUGAAGUAUGACCCCAGUAAUCCUGCUGCUGCCGCGGAGGCAAUGUUUGUUGAU
Rasp1-R1_Rep2 2801 UAAUCAUCGGAGACAUGCUUGUGUCCUUGUUCAGCGAAAGGAAGGGGUGAAGUAUGACCCCAGUAAUCCUGCUGCUGCCGCGGAGGCAAUGUUUGUUGAU
Rasp1-R1_Rep3 2801 UAAUCAUCGGAGACAUGCUUGUGUCCUUGUUCAGCGAAAGGAAGGGGUGAAGUAUGACCCCAGUAAUCCUGCUGCUGCCGCGGAGGCAAUGUUUGUUGAU

Rasp1-R1_2014 2901 AAUGAAACGCAGCAUCCACUGUCUGAGUGGAUGAAUAUGCAGGAGAUGAGUGCUGAGUUAUUGCUUAGGUAUCAACAACAUCGGGAGACUCAGCACGCAG
Rasp1-R1_Rep1 2901 AAUGAAACGCAGCAUCCACUGUCUGAGUGGAUGAAUAUGCAGGAGAUGAGUGCUGAGUUAUUGCUUAGGUAUCAACAACAUCGGGAGACUCAGCACGCAG
Rasp1-R1_Rep2 2901 AAUGAAACGCAGCAUCCACUGUCUGAGUGGAUGAAUAUGCAGGAGAUGAGUGCUGAGUUAUUGCUUAGGUAUCAACAACAUCGGGAGACUCAGCACGCAG
Rasp1-R1_Rep3 2901 AAUGAAACGCAGCAUCCACUGUCUGAGUGGAUGAAUAUGCAGGAGAUGAGUGCUGAGUUAUUGCUUAGGUAUCAACAACAUCGGGAGACUCAGCACGCAG

Rasp1-R1_2014 3001 AGUAUAGUUAUUGGAAGUCGACGUCUCGCUCUUCACAUGAUGUCUUUGACAUUUUGCAGAAGUGCGUAGAUGGUGAUGUCCAUUGGCUAUCUUUGCCCAU
Rasp1-R1_Rep1 3001 AGUAUAGUUAUUGGAAGUCGACGUCUCGCUCUUCACAUGAUGUCUUUGACAUUUUGCAGAAGUGCGUAGAUGGUGAUGUCCAUUGGCUAUCUUUGCCCAU
Rasp1-R1_Rep2 3001 AGUAUAGUUAUUGGAAGUCGACGUCUCGCUCUUCACAUGAUGUCUUUGACAUUUUGCAGAAGUGCGUAGAUGGUGAUGUCCAUUGGCUAUCUUUGCCCAU
Rasp1-R1_Rep3 3001 AGUAUAGUUAUUGGAAGUCGACGUCUCGCUCUUCACAUGAUGUCUUUGACAUUUUGCAGAAGUGCGUAGAUGGUGAUGUCCAUUGGCUAUCUUUGCCCAU

Rasp1-R1_2014 3101 UGACGUGAUACCCCCGACCAUUAGGCUCAAGUACAAGGGCAACCGAGUCUUUGCCAUUGAUGGUCGGACAUUUAUUUUCGACUACAUGACCCUAGAAUGU
Rasp1-R1_Rep1 3101 UGACGUGAUACCCCCGACCAUUAGGCUCAAGUACAAGGGCAACCGAGUCUUUGCCAUUGAUGGUCGGACAUUUAUUUUCGACUACAUGACCCUAGAAUGU
Rasp1-R1_Rep2 3101 UGACGUGAUACCCCCGACCAUUAGGCUCAAGUACAAGGGCAACCGAGUCUUUGCCAUUGAUGGUCGGACAUUUAUUUUCGACUACAUGACCCUAGAAUGU
Rasp1-R1_Rep3 3101 UGACGUGAUACCCCCGACCAUUAGGCUCAAGUACAAGGGCAACCGAGUCUUUGCCAUUGAUGGUCGGACAUUUAUUUUCGACUACAUGACCCUAGAAUGU

Rasp1-R1_2014 3201 GAAGAGAUUAAGGAGAAGAGCGAAAUUGAUGCUCGUCACCUUGAAUCUCGGAUUCUUGAGAAGUAUGGUGACACCCGCUUGCUUUUAGAAAAGUGGGGUG
Rasp1-R1_Rep1 3201 GAAGAGAUUAAGGAGAAGAGCGAAAUUGAUGCUCGUCACCUUGAAUCUCGGAUUCUUGAGAAGUAUGGUGACACCCGCUUGCUUUUAGAAAAGUGGGGUG
Rasp1-R1_Rep2 3201 GAAGAGAUUAAGGAGAAGAGCGAAAUUGAUGCUCGUCACCUUGAAUCUCGGAUUCUUGAGAAGUAUGGUGACACCCGCUUGCUUUUAGAAAAGUGGGGUG
Rasp1-R1_Rep3 3201 GAAGAGAUUAAGGAGAAGAGCGAAAUUGAUGCUCGUCACCUUGAAUCUCGGAUUCUUGAGAAGUAUGGUGACACCCGCUUGCUUUUAGAAAAGUGGGGUG

Rasp1-R1_2014 3301 CCAACGGCGUGGUAGCUCAGUUCAUUGAGCAACUCGUUGAGGGUCCGUCCAAUGUGGCAUCCAUGGAAGCCCUUUCAAAGGAUUCCUUGGAGAGCCAUAA
Rasp1-R1_Rep1 3301 CCAACGGCGUGGUAGCUCAGUUCAUUGAGCAACUCGUUGAGGGUCCGUCCAAUGUGGCAUCCAUGGAAGCCCUUUCAAAGGAUUCCUUGGAGAGCCAUAA
Rasp1-R1_Rep2 3301 CCAACGGCGUGGUAGCUCAGUUCAUUGAGCAACUCGUUGAGGGUCCGUCCAAUGUGGCAUCCAUGGAAGCCCUUUCAAAGGAUUCCUUGGAGAGCCAUAA
Rasp1-R1_Rep3 3301 CCAACGGCGUGGUAGCUCAGUUCAUUGAGCAACUCGUUGAGGGUCCGUCCAAUGUGGCAUCCAUGGAAGCCCUUUCAAAGGAUUCCUUGGAGAGCCAUAA

Rasp1-R1_2014 3401 AGAAUUUUUUUCAACUUUAGGUUUGAUUGAGAGGGCAACUCUCCGUGCUGUACAGAAGAAAAUAGAUAGUGCACGUGAGGAUUUGUCUUCUUUUGCAGGA
Rasp1-R1_Rep1 3401 AGAAUUUUUUUCAACUUUAGGUUUGAUUGAGAGGGCAACUCUCCGUGCUGUACAGAAGAAAAUAGAUAGUGCACGUGAGGAUUUGUCUUCUUUUGCAGGA
Rasp1-R1_Rep2 3401 AGAAUUUUUUUCAACUUUAGGUUUGAUUGAGAGGGCAACUCUCCGUGCUGUACAGAAGAAAAUAGAUAGUGCACGUGAGGAUUUGUCUUCUUUUGCAGGA
Rasp1-R1_Rep3 3401 AGAAUUUUUUUCAACUUUAGGUUUGAUUGAGAGGGCAACUCUCCGUGCUGUACAGAAGAAAAUAGAUAGUGCACGUGAGGAUUUGUCUUCUUUUGCAGGA

Rasp1-R1_2014 3501 UUGAAACCUGGUCGUUCGCUUGCAGAAUUGUUUGUAGAAGCGUAUGACUGGACUUACAAUCAUGGUGGUAAGCUUCUUUUAGUGCUUGCUGCUGUCAUUU
Rasp1-R1_Rep1 3501 UUGAAACCUGGUCGUUCGCUUGCAGAAUUGUUUGUAGAAGCGUAUGACUGGACUUACAAUCAUGGUGGUAAGCUUCUUUUAGUGCUUGCUGCUGUCAUUU
Rasp1-R1_Rep2 3501 UUGAAACCUGGUCGUUCGCUUGCAGAAUUGUUUGUAGAAGCGUAUGACUGGACUUACAAUCAUGGUGGUAAGCUUCUUUUAGUGCUUGCUGCUGUCAUUU
Rasp1-R1_Rep3 3501 UUGAAACCUGGUCGUUCGCUUGCAGAAUUGUUUGUAGAAGCGUAUGACUGGACUUACAAUCAUGGUGGUAAGCUUCUUUUAGUGCUUGCUGCUGUCAUUU

Synonymous mutation - Thr (ACU_3695_) -> Thr (ACC_3695_)

Rasp1-R1_2014 3601 UGAUUCUUUUUUUAGGUAGUGCGUGUGUUACUGCAAUGCGUGCAGUUUUCUGUGGCUCUGCUGCAGGAAGUGCAGUCGCUGUCGGAAGAAUGAC**U**GUUCA
Rasp1-R1_Rep1 3601 UGAUUCUUUUUUUAGGUAGUGCGUGUGUUACUGCAAUGCGUGCAGUUUUCUGUGGCUCUGCUGCAGGAAGUGCAGUCGCUGUCGGAAGAAUGAC**U**GUUCA
Rasp1-R1_Rep2 3601 UGAUUCUUUUUUUAGGUAGUGCGUGUGUUACUGCAAUGCGUGCAGUUUUCUGUGGCUCUGCUGCAGGAAGUGCAGUCGCUGUCGGAAGAAUGAC**U**GUUCA
Rasp1-R1_Rep3 3601 UGAUUCUUUUUUUAGGUAGUGCGUGUGUUACUGCAAUGCGUGCAGUUUUCUGUGGCUCUGCUGCAGGAAGUGCAGUCGCUGUCGGAAGAAUGAC**C**GUUCA

Rasp1-R1_2014 3701 AUCCACGAUUCCUUCCGGCAGUUACGCAGAUGUAUAUAAUGCUCGUAACAUGGCUCGCGUUUUCAGGCCGCAAUCUGUACAGAGUUCUUCUGCGGCCGAA
Rasp1-R1_Rep1 3701 AUCCACGAUUCCUUCCGGCAGUUACGCAGAUGUAUAUAAUGCUCGUAACAUGGCUCGCGUUUUCAGGCCGCAAUCUGUACAGAGUUCUUCUGCGGCCGAA
Rasp1-R1_Rep2 3701 AUCCACGAUUCCUUCCGGCAGUUACGCAGAUGUAUAUAAUGCUCGUAACAUGGCUCGCGUUUUCAGGCCGCAAUCUGUACAGAGUUCUUCUGCGGCCGAA
Rasp1-R1_Rep3 3701 AUCCACGAUUCCUUCCGGCAGUUACGCAGAUGUAUAUAAUGCUCGUAACAUGGCUCGCGUUUUCAGGCCGCAAUCUGUACAGAGUUCUUCUGCGGCCGAA

Rasp1-R1_2014 3801 GCUCAGUUCAAUGAAUCGCACGCCGUAAAUAUGUUGGUGCGAAUUGACCUCCCUGAUGGCAAUAUUAUUUCUGCCUGCAGGUUUCGUGGUAAGUCACUGG
Rasp1-R1_Rep1 3801 GCUCAGUUCAAUGAAUCGCACGCCGUAAAUAUGUUGGUGCGAAUUGACCUCCCUGAUGGCAAUAUUAUUUCUGCCUGCAGGUUUCGUGGUAAGUCACUGG
Rasp1-R1_Rep2 3801 GCUCAGUUCAAUGAAUCGCACGCCGUAAAUAUGUUGGUGCGAAUUGACCUCCCUGAUGGCAAUAUUAUUUCUGCCUGCAGGUUUCGUGGUAAGUCACUGG
Rasp1-R1_Rep3 3801 GCUCAGUUCAAUGAAUCGCACGCCGUAAAUAUGUUGGUGCGAAUUGACCUCCCUGAUGGCAAUAUUAUUUCUGCCUGCAGGUUUCGUGGUAAGUCACUGG

Rasp1-R1_2014 3901 CCCUGACGAAACAUCAGGCCUUGACAAUACCAACUGGAGCUAAGAUACAUAUUGUAUACACUGACAACAAUGGUAGUACUAAAGCUCCUUUGACACAUUA
Rasp1-R1_Rep1 3901 CCCUGACGAAACAUCAGGCCUUGACAAUACCAACUGGAGCUAAGAUACAUAUUGUAUACACUGACAACAAUGGUAGUACUAAAGCUCCUUUGACACAUUA
Rasp1-R1_Rep2 3901 CCCUGACGAAACAUCAGGCCUUGACAAUACCAACUGGAGCUAAGAUACAUAUUGUAUACACUGACAACAAUGGUAGUACUAAAGCUCCUUUGACACAUUA
Rasp1-R1_Rep3 3901 CCCUGACGAAACAUCAGGCCUUGACAAUACCAACUGGAGCUAAGAUACAUAUUGUAUACACUGACAACAAUGGUAGUACUAAAGCUCCUUUGACACAUUA

Rasp1-R1_2014 4001 UUUUCAACCUACGGGUCCCAAUGGAGAACAAUUUUUGAGAUUCUUCAACGGCACGGAGGUGUGUGUAUAUUCUCAUCCCCAGCUUUCCGCACUGCCAGGU
Rasp1-R1_Rep1 4001 UUUUCAACCUACGGGUCCCAAUGGAGAACAAUUUUUGAGAUUCUUCAACGGCACGGAGGUGUGUGUAUAUUCUCAUCCCCAGCUUUCCGCACUGCCAGGU
Rasp1-R1_Rep2 4001 UUUUCAACCUACGGGUCCCAAUGGAGAACAAUUUUUGAGAUUCUUCAACGGCACGGAGGUGUGUGUAUAUUCUCAUCCCCAGCUUUCCGCACUGCCAGGU
Rasp1-R1_Rep3 4001 UUUUCAACCUACGGGUCCCAAUGGAGAACAAUUUUUGAGAUUCUUCAACGGCACGGAGGUGUGUGUAUAUUCUCAUCCCCAGCUUUCCGCACUGCCAGGU

Rasp1-R1_2014 4101 GCUCCACAAAAUUACUUUUUGAAAGAUGUGGAGAAAAUUACUGGUGACAUAGCUAUUAAAGGCUGUGGCAUUAAAUUGGGCCGGACUAGUGUUGGCAAUU
Rasp1-R1_Rep1 4101 GCUCCACAAAAUUACUUUUUGAAAGAUGUGGAGAAAAUUACUGGUGACAUAGCUAUUAAAGGCUGUGGCAUUAAAUUGGGCCGGACUAGUGUUGGCAAUU
Rasp1-R1_Rep2 4101 GCUCCACAAAAUUACUUUUUGAAAGAUGUGGAGAAAAUUACUGGUGACAUAGCUAUUAAAGGCUGUGGCAUUAAAUUGGGCCGGACUAGUGUUGGCAAUU
Rasp1-R1_Rep3 4101 GCUCCACAAAAUUACUUUUUGAAAGAUGUGGAGAAAAUUACUGGUGACAUAGCUAUUAAAGGCUGUGGCAUUAAAUUGGGCCGGACUAGUGUUGGCAAUU

Rasp1-R1_2014 4201 GUGUCGGUAUCCAGAGCAAUGAACCUGUUCUGAAUCACUGGCGUGCUGUUGCGCGAGUUCGCACCACCAAGGUCACAAUUGAUAAUUAUGUUGAUGGUGG
Rasp1-R1_Rep1 4201 GUGUCGGUAUCCAGAGCAAUGAACCUGUUCUGAAUCACUGGCGUGCUGUUGCGCGAGUUCGCACCACCAAGGUCACAAUUGAUAAUUAUGUUGAUGGUGG
Rasp1-R1_Rep2 4201 GUGUCGGUAUCCAGAGCAAUGAACCUGUUCUGAAUCACUGGCGUGCUGUUGCGCGAGUUCGCACCACCAAGGUCACAAUUGAUAAUUAUGUUGAUGGUGG
Rasp1-R1_Rep3 4201 GUGUCGGUAUCCAGAGCAAUGAACCUGUUCUGAAUCACUGGCGUGCUGUUGCGCGAGUUCGCACCACCAAGGUCACAAUUGAUAAUUAUGUUGAUGGUGG

Rasp1-R1_2014 4301 UGAUUACGUCAACGACCUACCCACAUCGCUUAUUUCUGAGUAUGUUAAUUCGCCGGAGGAUUGUGGCGCGCUUUUGGUCGCCCAUCUGGAGGGUGGUUAC
Rasp1-R1_Rep1 4301 UGAUUACGUCAACGACCUACCCACAUCGCUUAUUUCUGAGUAUGUUAAUUCGCCGGAGGAUUGUGGCGCGCUUUUGGUCGCCCAUCUGGAGGGUGGUUAC
Rasp1-R1_Rep2 4301 UGAUUACGUCAACGACCUACCCACAUCGCUUAUUUCUGAGUAUGUUAAUUCGCCGGAGGAUUGUGGCGCGCUUUUGGUCGCCCAUCUGGAGGGUGGUUAC
Rasp1-R1_Rep3 4301 UGAUUACGUCAACGACCUACCCACAUCGCUUAUUUCUGAGUAUGUUAAUUCGCCGGAGGAUUGUGGCGCGCUUUUGGUCGCCCAUCUGGAGGGUGGUUAC

Rasp1-R1_2014 4401 AAAAUCAUUGGUAUGCAUGUGGCAGGAUCUUCUUAUCCUGUGGAAGUUGAUGGAGUCCAAGUGCCGCGAUACAUUUCGCACGCGGCCUUCUUCCCUGAUU
Rasp1-R1_Rep1 4401 AAAAUCAUUGGUAUGCAUGUGGCAGGAUCUUCUUAUCCUGUGGAAGUUGAUGGAGUCCAAGUGCCGCGAUACAUUUCGCACGCGGCCUUCUUCCCUGAUU
Rasp1-R1_Rep2 4401 AAAAUCAUUGGUAUGCAUGUGGCAGGAUCUUCUUAUCCUGUGGAAGUUGAUGGAGUCCAAGUGCCGCGAUACAUUUCGCACGCGGCCUUCUUCCCUGAUU
Rasp1-R1_Rep3 4401 AAAAUCAUUGGUAUGCAUGUGGCAGGAUCUUCUUAUCCUGUGGAAGUUGAUGGAGUCCAAGUGCCGCGAUACAUUUCGCACGCGGCCUUCUUCCCUGAUU

Rasp1-R1_2014 4501 UUUCCUCCUUUGCCCCUUGCCAGUCUAGCAUUAUCAAGUCCCUUGUUCAGGAAGCUGGCAUUGAGGAGAGAGGAGUGUCUAAAGUGGGGCAUAUUAAGGA
Rasp1-R1_Rep1 4501 UUUCCUCCUUUGCCCCUUGCCAGUCUAGCAUUAUCAAGUCCCUUGUUCAGGAAGCUGGCAUUGAGGAGAGAGGAGUGUCUAAAGUGGGGCAUAUUAAGGA
Rasp1-R1_Rep2 4501 UUUCCUCCUUUGCCCCUUGCCAGUCUAGCAUUAUCAAGUCCCUUGUUCAGGAAGCUGGCAUUGAGGAGAGAGGAGUGUCUAAAGUGGGGCAUAUUAAGGA
Rasp1-R1_Rep3 4501 UUUCCUCCUUUGCCCCUUGCCAGUCUAGCAUUAUCAAGUCCCUUGUUCAGGAAGCUGGCAUUGAGGAGAGAGGAGUGUCUAAAGUGGGGCAUAUUAAGGA

Rasp1-R1_2014 4601 UCCUGCUGAGACACCACAUGUGGGAGGGAAAACUAAGCUUGAGUUGGUGGAUGAAGCCUUUUUGGUGCCAUCACCAGUUGAAGUUAAGAUCCCUUCUAUU
Rasp1-R1_Rep1 4601 UCCUGCUGAGACACCACAUGUGGGAGGGAAAACUAAGCUUGAGUUGGUGGAUGAAGCCUUUUUGGUGCCAUCACCAGUUGAAGUUAAGAUCCCUUCUAUU
Rasp1-R1_Rep2 4601 UCCUGCUGAGACACCACAUGUGGGAGGGAAAACUAAGCUUGAGUUGGUGGAUGAAGCCUUUUUGGUGCCAUCACCAGUUGAAGUUAAGAUCCCUUCUAUU
Rasp1-R1_Rep3 4601 UCCUGCUGAGACACCACAUGUGGGAGGGAAAACUAAGCUUGAGUUGGUGGAUGAAGCCUUUUUGGUGCCAUCACCAGUUGAAGUUAAGAUCCCUUCUAUU

Rasp1-R1_2014 4701 CUUUCAAAGGAUGAUCCUCGCAUUCCCGAAGCUUACAAGGGAUAUGAUCCAUUGGGCGAUGCUAUGGAAAAAUUUUACGAGCCCAUGCUGGACCUCGAUG
Rasp1-R1_Rep1 4701 CUUUCAAAGGAUGAUCCUCGCAUUCCCGAAGCUUACAAGGGAUAUGAUCCAUUGGGCGAUGCUAUGGAAAAAUUUUACGAGCCCAUGCUGGACCUCGAUG
Rasp1-R1_Rep2 4701 CUUUCAAAGGAUGAUCCUCGCAUUCCCGAAGCUUACAAGGGAUAUGAUCCAUUGGGCGAUGCUAUGGAAAAAUUUUACGAGCCCAUGCUGGACCUCGAUG
Rasp1-R1_Rep3 4701 CUUUCAAAGGAUGAUCCUCGCAUUCCCGAAGCUUACAAGGGAUAUGAUCCAUUGGGCGAUGCUAUGGAAAAAUUUUACGAGCCCAUGCUGGACCUCGAUG

 Synonymous mutation - Tyr (UAU_4835_) -> Tyr (UAC_4835_)

Rasp1-R1_2014 4801 GGGAUGUUUUGGAGUGCGUUAUGGCUGAUAUGUA**U**GACGAGUUCUAUGAUUGCCAGACGACACUUCGCAUUAUGUCUGAUGAUGAAGUCAUUAAUGGCAG
Rasp1-R1_Rep1 4801 GGGAUGUUUUGGAGUGCGUUAUGGCUGAUAUGUA**C**GACGAGUUCUAUGAUUGCCAGACGACACUUCGCAUUAUGUCUGAUGAUGAAGUCAUUAAUGGCAG
Rasp1-R1_Rep2 4801 GGGAUGUUUUGGAGUGCGUUAUGGCUGAUAUGUA**C**GACGAGUUCUAUGAUUGCCAGACGACACUUCGCAUUAUGUCUGAUGAUGAAGUCAUUAAUGGCAG
Rasp1-R1_Rep3 4801 GGGAUGUUUUGGAGUGCGUUAUGGCUGAUAUGUA**C**GACGAGUUCUAUGAUUGCCAGACGACACUUCGCAUUAUGUCUGAUGAUGAAGUCAUUAAUGGCAG

Rasp1-R1_2014 4901 CGACUUUGGUUUUAAUAUCGAGGCCGUUGUUAAAGGGACUUCUGAAGGCUACCCGUUUGUUUUGAGUCGACGAGCGGGUGAAAAAGGUAAAGCUCGUUUU
Rasp1-R1_Rep1 4901 CGACUUUGGUUUUAAUAUCGAGGCCGUUGUUAAAGGGACUUCUGAAGGCUACCCGUUUGUUUUGAGUCGACGAGCGGGUGAAAAAGGUAAAGCUCGUUUU
Rasp1-R1_Rep2 4901 CGACUUUGGUUUUAAUAUCGAGGCCGUUGUUAAAGGGACUUCUGAAGGCUACCCGUUUGUUUUGAGUCGACGAGCGGGUGAAAAAGGUAAAGCUCGUUUU
Rasp1-R1_Rep3 4901 CGACUUUGGUUUUAAUAUCGAGGCCGUUGUUAAAGGGACUUCUGAAGGCUACCCGUUUGUUUUGAGUCGACGAGCGGGUGAAAAAGGUAAAGCUCGUUUU

Rasp1-R1_2014 5001 UUAGAAGAGCUAGAGCCUCAACCAGGAGACACCAAGCCCAAAUACAAGUUGGUUGAAGGUACCGAGGUGCAUUCCGCUAUGAUGGCAAUGGAGGAGCAGG
Rasp1-R1_Rep1 5001 UUAGAAGAGCUAGAGCCUCAACCAGGAGACACCAAGCCCAAAUACAAGUUGGUUGAAGGUACCGAGGUGCAUUCCGCUAUGAUGGCAAUGGAGGAGCAGG
Rasp1-R1_Rep2 5001 UUAGAAGAGCUAGAGCCUCAACCAGGAGACACCAAGCCCAAAUACAAGUUGGUUGAAGGUACCGAGGUGCAUUCCGCUAUGAUGGCAAUGGAGGAGCAGG
Rasp1-R1_Rep3 5001 UUAGAAGAGCUAGAGCCUCAACCAGGAGACACCAAGCCCAAAUACAAGUUGGUUGAAGGUACCGAGGUGCAUUCCGCUAUGAUGGCAAUGGAGGAGCAGG

Rasp1-R1_2014 5101 CUCGUACUGAGGUUCCGUUGCUUAUUGGAAUGGAUGUUCCGAAGGAUGAGAGACUCAAGCCGUCCAAGGUACUGGAGAAGCCUAAGACGCGCACAUUCGU
Rasp1-R1_Rep1 5101 CUCGUACUGAGGUUCCGUUGCUUAUUGGAAUGGAUGUUCCGAAGGAUGAGAGACUCAAGCCGUCCAAGGUACUGGAGAAGCCUAAGACGCGCACAUUCGU
Rasp1-R1_Rep2 5101 CUCGUACUGAGGUUCCGUUGCUUAUUGGAAUGGAUGUUCCGAAGGAUGAGAGACUCAAGCCGUCCAAGGUACUGGAGAAGCCUAAGACGCGCACAUUCGU
Rasp1-R1_Rep3 5101 CUCGUACUGAGGUUCCGUUGCUUAUUGGAAUGGAUGUUCCGAAGGAUGAGAGACUCAAGCCGUCCAAGGUACUGGAGAAGCCUAAGACGCGCACAUUCGU

Rasp1-R1_2014 5201 UGUUCUCCCAAUGCACUAUAACUUGUUGCUACGCAAGUACGUAGGAAUACUUUGUUCUAGCAUGCAAGUUAAUAGGCACCGUUUGGCAUGCGCUGUGGGA
Rasp1-R1_Rep1 5201 UGUUCUCCCAAUGCACUAUAACUUGUUGCUACGCAAGUACGUAGGAAUACUUUGUUCUAGCAUGCAAGUUAAUAGGCACCGUUUGGCAUGCGCUGUGGGA
Rasp1-R1_Rep2 5201 UGUUCUCCCAAUGCACUAUAACUUGUUGCUACGCAAGUACGUAGGAAUACUUUGUUCUAGCAUGCAAGUUAAUAGGCACCGUUUGGCAUGCGCUGUGGGA
Rasp1-R1_Rep3 5201 UGUUCUCCCAAUGCACUAUAACUUGUUGCUACGCAAGUACGUAGGAAUACUUUGUUCUAGCAUGCAAGUUAAUAGGCACCGUUUGGCAUGCGCUGUGGGA

Rasp1-R1_2014 5301 ACCAACCCAUAUUCGCGUGAUUGGACGGAUAUUUAUCAACGCCUAGCUGAAAAAAAUUCAGUAGCCUUGAACUGCGACUAUAGUCGUUUUGAUGGGCUAC
Rasp1-R1_Rep1 5301 ACCAACCCAUAUUCGCGUGAUUGGACGGAUAUUUAUCAACGCCUAGCUGAAAAAAAUUCAGUAGCCUUGAACUGCGACUAUAGUCGUUUUGAUGGGCUAC
Rasp1-R1_Rep2 5301 ACCAACCCAUAUUCGCGUGAUUGGACGGAUAUUUAUCAACGCCUAGCUGAAAAAAAUUCAGUAGCCUUGAACUGCGACUAUAGUCGUUUUGAUGGGCUAC
Rasp1-R1_Rep3 5301 ACCAACCCAUAUUCGCGUGAUUGGACGGAUAUUUAUCAACGCCUAGCUGAAAAAAAUUCAGUAGCCUUGAACUGCGACUAUAGUCGUUUUGAUGGGCUAC

Rasp1-R1_2014 5401 UUAAUUAUCAGGCAUAUGUUCAUAUUGUAAAUUUUAUUAAUAGGUUAUAUAAUGAUGAACAUAGUACCGUUCGUGGCAAUCUUUUAAUGGCCAUGUAUGG
Rasp1-R1_Rep1 5401 UUAAUUAUCAGGCAUAUGUUCAUAUUGUAAAUUUUAUUAAUAGGUUAUAUAAUGAUGAACAUAGUACCGUUCGUGGCAAUCUUUUAAUGGCCAUGUAUGG
Rasp1-R1_Rep2 5401 UUAAUUAUCAGGCAUAUGUUCAUAUUGUAAAUUUUAUUAAUAGGUUAUAUAAUGAUGAACAUAGUACCGUUCGUGGCAAUCUUUUAAUGGCCAUGUAUGG
Rasp1-R1_Rep3 5401 UUAAUUAUCAGGCAUAUGUUCAUAUUGUAAAUUUUAUUAAUAGGUUAUAUAAUGAUGAACAUAGUACCGUUCGUGGCAAUCUUUUAAUGGCCAUGUAUGG

Rasp1-R1_2014 5501 UAGGUGGAGUGUGUGUGGACAACGUGUUUACGAAGUUCGCGCUGGCAUGCCCUCUGGAUGUGCCCUCACCGUGAUUAUUAACUCUCUUUUUAACGAGCUA
Rasp1-R1_Rep1 5501 UAGGUGGAGUGUGUGUGGACAACGUGUUUACGAAGUUCGCGCUGGCAUGCCCUCUGGAUGUGCCCUCACCGUGAUUAUUAACUCUCUUUUUAACGAGCUA
Rasp1-R1_Rep2 5501 UAGGUGGAGUGUGUGUGGACAACGUGUUUACGAAGUUCGCGCUGGCAUGCCCUCUGGAUGUGCCCUCACCGUGAUUAUUAACUCUCUUUUUAACGAGCUA
Rasp1-R1_Rep3 5501 UAGGUGGAGUGUGUGUGGACAACGUGUUUACGAAGUUCGCGCUGGCAUGCCCUCUGGAUGUGCCCUCACCGUGAUUAUUAACUCUCUUUUUAACGAGCUA

Rasp1-R1_2014 5601 UUAAUCAGAUAUGUUUAUCGCGUCACUGUACCACGCUCCCUAGUAAAUAAUUUUAAACAGGAAGUGUGUCUCAUAGUAUACGGUGACGAUAAUUUGAUUU
Rasp1-R1_Rep1 5601 UUAAUCAGAUAUGUUUAUCGCGUCACUGUACCACGCUCCCUAGUAAAUAAUUUUAAACAGGAAGUGUGUCUCAUAGUAUACGGUGACGAUAAUUUGAUUU
Rasp1-R1_Rep2 5601 UUAAUCAGAUAUGUUUAUCGCGUCACUGUACCACGCUCCCUAGUAAAUAAUUUUAAACAGGAAGUGUGUCUCAUAGUAUACGGUGACGAUAAUUUGAUUU
Rasp1-R1_Rep3 5601 UUAAUCAGAUAUGUUUAUCGCGUCACUGUACCACGCUCCCUAGUAAAUAAUUUUAAACAGGAAGUGUGUCUCAUAGUAUACGGUGACGAUAAUUUGAUUU

Rasp1-R1_2014 5701 CUAUUAAGCCAGACACCAUGAAAUAUUUCAAUGGUGAACAGAUUAAAUCUACUCUGGCUAAAUAUAGGAUUACCAUUACUGAUGGCAGUGAUAAGAACUC
Rasp1-R1_Rep1 5701 CUAUUAAGCCAGACACCAUGAAAUAUUUCAAUGGUGAACAGAUUAAAUCUACUCUGGCUAAAUAUAGGAUUACCAUUACUGAUGGCAGUGAUAAGAACUC
Rasp1-R1_Rep2 5701 CUAUUAAGCCAGACACCAUGAAAUAUUUCAAUGGUGAACAGAUUAAAUCUACUCUGGCUAAAUAUAGGAUUACCAUUACUGAUGGCAGUGAUAAGAACUC
Rasp1-R1_Rep3 5701 CUAUUAAGCCAGACACCAUGAAAUAUUUCAAUGGUGAACAGAUUAAAUCUACUCUGGCUAAAUAUAGGAUUACCAUUACUGAUGGCAGUGAUAAGAACUC

Missense mutation - Lys (AA_5812_A) -> Arg (AG_5812_A)

Rasp1-R1_2014 5801 GCCAGUUCUUA**A**AGCCAAGCCUCUGAAGCAACUUGAUUUUUUGAAGAGAGGCUUUAGGGUUGAAAGUGAUGGAAGGGUGCUUGCCCCCCUCGAUUUGCAA
Rasp1-R1_Rep1 5801 GCCAGUUCUUA**G**AGCCAAGCCUCUGAAGCAACUUGAUUUUUUGAAGAGAGGCUUUAGGGUUGAAAGUGAUGGAAGGGUGCUUGCCCCCCUCGAUUUGCAA
Rasp1-R1_Rep2 5801 GCCAGUUCUUA**G**AGCCAAGCCUCUGAAGCAACUUGAUUUUUUGAAGAGAGGCUUUAGGGUUGAAAGUGAUGGAAGGGUGCUUGCCCCCCUCGAUUUGCAA
Rasp1-R1_Rep3 5801 GCCAGUUCUUA**G**AGCCAAGCCUCUGAAGCAACUUGAUUUUUUGAAGAGAGGCUUUAGGGUUGAAAGUGAUGGAAGGGUGCUUGCCCCCCUCGAUUUGCAA

Rasp1-R1_2014 5901 GCUAUCUAUUCUUCCCUGUAUUAUAUUAACCCACAGGGAAACAUAUUAAAUUCUUUAUUUUUAAAUGCUCAGGUUGCUCUGAGAGAGUUGUAUCUCCAUG
Rasp1-R1_Rep1 5901 GCUAUCUAUUCUUCCCUGUAUUAUAUUAACCCACAGGGAAACAUAUUAAAUUCUUUAUUUUUAAAUGCUCAGGUUGCUCUGAGAGAGUUGUAUCUCCAUG
Rasp1-R1_Rep2 5901 GCUAUCUAUUCUUCCCUGUAUUAUAUUAACCCACAGGGAAACAUAUUAAAUUCUUUAUUUUUAAAUGCUCAGGUUGCUCUGAGAGAGUUGUAUCUCCAUG
Rasp1-R1_Rep3 5901 GCUAUCUAUUCUUCCCUGUAUUAUAUUAACCCACAGGGAAACAUAUUAAAUUCUUUAUUUUUAAAUGCUCAGGUUGCUCUGAGAGAGUUGUAUCUCCAUG

Rasp1-R1_2014 6001 GUGAUGUUGAGCAAUUCACUGCUGUCAGGAAUUUCUACGUCAAGCAAAUUGGCGGGAACUUCCUGAAUUUACCCCAAUGGAGACACUGUGCUUCCUUUCA
Rasp1-R1_Rep1 6001 GUGAUGUUGAGCAAUUCACUGCUGUCAGGAAUUUCUACGUCAAGCAAAUUGGCGGGAACUUCCUGAAUUUACCCCAAUGGAGACACUGUGCUUCCUUUCA
Rasp1-R1_Rep2 6001 GUGAUGUUGAGCAAUUCACUGCUGUCAGGAAUUUCUACGUCAAGCAAAUUGGCGGGAACUUCCUGAAUUUACCCCAAUGGAGACACUGUGCUUCCUUUCA
Rasp1-R1_Rep3 6001 GUGAUGUUGAGCAAUUCACUGCUGUCAGGAAUUUCUACGUCAAGCAAAUUGGCGGGAACUUCCUGAAUUUACCCCAAUGGAGACACUGUGCUUCCUUUCA

Rasp1-R1_2014 6101 UGAUGAGCAGUAUUCUCAAUGGAAGCCGUGGUCACCUGUGAAAUUUUUGGAGGUAGAUGUGCCUGAUGUUAAAUUUUUGCAACACAAGGCACCAGCCACC
Rasp1-R1_Rep1 6101 UGAUGAGCAGUAUUCUCAAUGGAAGCCGUGGUCACCUGUGAAAUUUUUGGAGGUAGAUGUGCCUGAUGUUAAAUUUUUGCAACACAAGGCACCAGCCACC
Rasp1-R1_Rep2 6101 UGAUGAGCAGUAUUCUCAAUGGAAGCCGUGGUCACCUGUGAAAUUUUUGGAGGUAGAUGUGCCUGAUGUUAAAUUUUUGCAACACAAGGCACCAGCCACC
Rasp1-R1_Rep3 6101 UGAUGAGCAGUAUUCUCAAUGGAAGCCGUGGUCACCUGUGAAAUUUUUGGAGGUAGAUGUGCCUGAUGUUAAAUUUUUGCAACACAAGGCACCAGCCACC

Rasp1-R1_2014 6201 GCCCUUUCGAUUGUUGCUGAUAGACUCGCGGUUGCAGGACCUGGAUGGCGCAAUAAAGAUCCAGAUAAGUACUUGCUUGUGAGUCUUACUAGUUUAAAGG
Rasp1-R1_Rep1 6201 GCCCUUUCGAUUGUUGCUGAUAGACUCGCGGUUGCAGGACCUGGAUGGCGCAAUAAAGAUCCAGAUAAGUACUUGCUUGUGAGUCUUACUAGUUUAAAGG
Rasp1-R1_Rep2 6201 GCCCUUUCGAUUGUUGCUGAUAGACUCGCGGUUGCAGGACCUGGAUGGCGCAAUAAAGAUCCAGAUAAGUACUUGCUUGUGAGUCUUACUAGUUUAAAGG
Rasp1-R1_Rep3 6201 GCCCUUUCGAUUGUUGCUGAUAGACUCGCGGUUGCAGGACCUGGAUGGCGCAAUAAAGAUCCAGAUAAGUACUUGCUUGUGAGUCUUACUAGUUUAAAGG

Rasp1-R1_2014 6301 CUAACGAGGGCGGCCUGUAUUUUCCCGUUGAUUAUGGGGAAGGUACAGGACAACAAGCUACGGAAGCUUCUAUUAGAGCUUACCGUAGAUUAAAAGAUCA
Rasp1-R1_Rep1 6301 CUAACGAGGGCGGCCUGUAUUUUCCCGUUGAUUAUGGGGAAGGUACAGGACAACAAGCUACGGAAGCUUCUAUUAGAGCUUACCGUAGAUUAAAAGAUCA
Rasp1-R1_Rep2 6301 CUAACGAGGGCGGCCUGUAUUUUCCCGUUGAUUAUGGGGAAGGUACAGGACAACAAGCUACGGAAGCUUCUAUUAGAGCUUACCGUAGAUUAAAAGAUCA
Rasp1-R1_Rep3 6301 CUAACGAGGGCGGCCUGUAUUUUCCCGUUGAUUAUGGGGAAGGUACAGGACAACAAGCUACGGAAGCUUCUAUUAGAGCUUACCGUAGAUUAAAAGAUCA

Rasp1-R1_2014 6401 UCGCGUACGCCAUAUGCGCGAUUCUUGGAAUGAAGGGAAAACCAUCGUGUUUCGAUGUGAGGGUCCUUUCGUUUCUGGUUGGGCUGCAGCCAUCUCGUUC
Rasp1-R1_Rep1 6401 UCGCGUACGCCAUAUGCGCGAUUCUUGGAAUGAAGGGAAAACCAUCGUGUUUCGAUGUGAGGGUCCUUUCGUUUCUGGUUGGGCUGCAGCCAUCUCGUUC
Rasp1-R1_Rep2 6401 UCGCGUACGCCAUAUGCGCGAUUCUUGGAAUGAAGGGAAAACCAUCGUGUUUCGAUGUGAGGGUCCUUUCGUUUCUGGUUGGGCUGCAGCCAUCUCGUUC
Rasp1-R1_Rep3 6401 UCGCGUACGCCAUAUGCGCGAUUCUUGGAAUGAAGGGAAAACCAUCGUGUUUCGAUGUGAGGGUCCUUUCGUUUCUGGUUGGGCUGCAGCCAUCUCGUUC

Rasp1-R1_2014 6501 GGUGCGAGUAUUGGUAUGAAUGCCCAAGAUCUACUCAUCAAUUAUGGCAUACAAGGUGGCGCUCAUAGAGAUUUUCUAGGGCGCUAUUUUAUUAACACAC
Rasp1-R1_Rep1 6501 GGUGCGAGUAUUGGUAUGAAUGCCCAAGAUCUACUCAUCAAUUAUGGCAUACAAGGUGGCGCUCAUAGAGAUUUUCUAGGGCGCUAUUUUAUUAACACAC
Rasp1-R1_Rep2 6501 GGUGCGAGUAUUGGUAUGAAUGCCCAAGAUCUACUCAUCAAUUAUGGCAUACAAGGUGGCGCUCAUAGAGAUUUUCUAGGGCGCUAUUUUAUUAACACAC
Rasp1-R1_Rep3 6501 GGUGCGAGUAUUGGUAUGAAUGCCCAAGAUCUACUCAUCAAUUAUGGCAUACAAGGUGGCGCUCAUAGAGAUUUUCUAGGGCGCUAUUUUAUUAACACAC

Rasp1-R1_2014 6601 GCUUCAAAGAGCUGGAGCGUUAUGAUAGGCCUUAUGCCACUCGCAUUUCAGCGGGCUGAGGUUUUUUUAGGUUAGGCGAGUAGCUGCCGUAAGCAGCUUC
Rasp1-R1_Rep1 6601 GCUUCAAAGAGCUGGAGCGUUAUGAUAGGCCUUAUGCCACUCGCAUUUCAGCGGGCUGAGGUUUUUUUAGGUUAGGCGAGUAGCUGCCGUAAGCAGCUUC
Rasp1-R1_Rep2 6601 GCUUCAAAGAGCUGGAGCGUUAUGAUAGGCCUUAUGCCACUCGCAUUUCAGCGGGCUGAGGUUUUUUUAGGUUAGGCGAGUAGCUGCCGUAAGCAGCUUC
Rasp1-R1_Rep3 6601 GCUUCAAAGAGCUGGAGCGUUAUGAUAGGCCUUAUGCCACUCGCAUUUCAGCGGGCUGAGGUUUUUUUAGGUUAGGCGAGUAGCUGCCGUAAGCAGCUUC

Rasp1-R1_2014 6701 CAAUAGGUGGCCUCUUAAUUAGCUUUGAAAUAGGGGUUAUCUAGCCUUGAGCAAGCUGGCACCGGUCCUGAUGGACUACCAGGAAAGUACCUGGUUUUGG
Rasp1-R1_Rep1 6701 CAAUAGGUGGCCUCUUAAUUAGCUUUGAAAUAGGGGUUAUCUAGCCUUGAGCAAGCUGGCACCGGUCCUGAUGGACUACCAGGAAAGUACCUGGUUUUGG
Rasp1-R1_Rep2 6701 CAAUAGGUGGCCUCUUAAUUAGCUUUGAAAUAGGGGUUAUCUAGCCUUGAGCAAGCUGGCACCGGUCCUGAUGGACUACCAGGAAAGUACCUGGUUUUGG
Rasp1-R1_Rep3 6701 CAAUAGGUGGCCUCUUAAUUAGCUUUGAAAUAGGGGUUAUCUAGCCUUGAGCAAGCUGGCACCGGUCCUGAUGGACUACCAGGAAAGUACCUGGUUUUGG

Rasp1-R1_2014 6801 AAGAAUUUGUAGUGGGAUUCUUAAAUCUUGCCUGCUACUAGGAGCCUUUAGUAAGCUCUGAAUCUAUUUACUCCCAAAAGGUGAAGCGAAACUAGCCAUC
Rasp1-R1_Rep1 6801 AAGAAUUUGUAGUGGGAUUCUUAAAUCUUGCCUGCUACUAGGAGCCUUUAGUAAGCUCUGAAUCUAUUUACUCCCAAAAGGUGAAGCGAAACUAGCCAUC
Rasp1-R1_Rep2 6801 AAGAAUUUGUAGUGGGAUUCUUAAAUCUUGCCUGCUACUAGGAGCCUUUAGUAAGCUCUGAAUCUAUUUACUCCCAAAAGGUGAAGCGAAACUAGCCAUC
Rasp1-R1_Rep3 6801 AAGAAUUUGUAGUGGGAUUCUUAAAUCUUGCCUGCUACUAGGAGCCUUUAGUAAGCUCUGAAUCUAUUUACUCCCAAAAGGUGAAGCGAAACUAGCCAUC

Mutation at 3’ UTR (G_6907_) -> (A_6907_)

Rasp1-R1_2014 6901 UCCUUG**G**AAAAGGUGUGUGUAAGUGCUUAAUUUCCUUCGGGCCGUUGUGGAUCCUAUAACGGAGUUUGUAGAAUAUAUGACACUAGGAAAGACUAGCGUU
Rasp1-R1_Rep1 6901 UCCUUG**G**AAAAGGUGUGUGUAAGUGCUUAAUUUCCUUCGGGCCGUUGUGGAUCCUAUAACGGAGUUUGUAGAAUAUAUGACACUAGGAAAGACUAGCGUU
Rasp1-R1_Rep2 6901 UCCUUG**A**AAAAGGUGUGUGUAAGUGCUUAAUUUCCUUCGGGCCGUUGUGGAUCCUAUAACGGAGUUUGUAGAAUAUAUGACACUAGGAAAGACUAGCGUU
Rasp1-R1_Rep3 6901 UCCUUG**A**AAAAGGUGUGUGUAAGUGCUUAAUUUCCUUCGGGCCGUUGUGGAUCCUAUAACGGAGUUUGUAGAAUAUAUGACACUAGGAAAGACUAGCGUU

Rasp1-R1_2014 7001 UUAUAGUGAUUGACGUAAAUAAUCACUAAGGACACCUGGAGAGACAGGCGCUUUGGCAGCUCAUGCGUUAUCGACGCUGCUACCUUUAUUUGUUUGGGGA
Rasp1-R1_Rep1 7001 UUAUAGUGAUUGACGUAAAUAAUCACUAAGGACACCUGGAGAGACAGGCGCUUUGGCAGCUCAUGCGUUAUCGACGCUGCUACCUUUAUUUGUUUGGGGA
Rasp1-R1_Rep2 7001 UUAUAGUGAUUGACGUAAAUAAUCACUAAGGACACCUGGAGAGACAGGCGCUUUGGCAGCUCAUGCGUUAUCGACGCUGCUACCUUUAUUUGUUUGGGGA
Rasp1-R1_Rep3 7001 UUAUAGUGAUUGACGUAAAUAAUCACUAAGGACACCUGGAGAGACAGGCGCUUUGGCAGCUCAUGCGUUAUCGACGCUGCUACCUUUAUUUGUUUGGGGA

Rasp1-R1_2014 7101 AACAAACAUCAUAUCUUGAGUCUGCAUUCAAAUUUAUAAUAAUGUAGUUGUCAUAGCCUACCGAUGAGCCUGCGAGAAAGGUUCCAUGAAGACUAGAGUU
Rasp1-R1_Rep1 7101 AACAAACAUCAUAUCUUGAGUCUGCAUUCAAAUUUAUAAUAAUGUAGUUGUCAUAGCCUACCGAUGAGCCUGCGAGAAAGGUUCCAUGAAGACUAGAGUU
Rasp1-R1_Rep2 7101 AACAAACAUCAUAUCUUGAGUCUGCAUUCAAAUUUAUAAUAAUGUAGUUGUCAUAGCCUACCGAUGAGCCUGCGAGAAAGGUUCCAUGAAGACUAGAGUU
Rasp1-R1_Rep3 7101 AACAAACAUCAUAUCUUGAGUCUGCAUUCAAAUUUAUAAUAAUGUAGUUGUCAUAGCCUACCGAUGAGCCUGCGAGAAAGGUUCCAUGAAGACUAGAGUU

Rasp1-R1_2014 7201 GGCUAACUCCCACUUAAUCUCUCUAUUGAUCAUUCGACAGUGUGUUGAGAACUUAUGGGUUUUAUCACCUAUGGUGGAGCGGUUGCAACCCAACUGUAGA
Rasp1-R1_Rep1 7201 GGCUAACUCCCACUUAAUCUCUCUAUUGAUCAUUCGACAGUGUGUUGAGAACUUAUGGGUUUUAUCACCUAUGGUGGAGCGGUUGCAACCCAACUGUAGA
Rasp1-R1_Rep2 7201 GGCUAACUCCCACUUAAUCUCUCUAUUGAUCAUUCGACAGUGUGUUGAGAACUUAUGGGUUUUAUCACCUAUGGUGGAGCGGUUGCAACCCAACUGUAGA
Rasp1-R1_Rep3 7201 GGCUAACUCCCACUUAAUCUCUCUAUUGAUCAUUCGACAGUGUGUUGAGAACUUAUGGGUUUUAUCACCUAUGGUGGAGCGGUUGCAACCCAACUGUAGA

Mutation at 3’ UTR (G_7384_) -> (A_7384_)

Rasp1-R1_2014 7301 UGUCACUUGGAUUGAGGCACUCACCACGCUUCAUUUCAAGUAAAGUAUCGAUUCGUCGGUAUGAUUCUCCAUUAUAGCUCUUG**G**UAAACGGUAAGUUCAU
Rasp1-R1_Rep1 7301 UGUCACUUGGAUUGAGGCACUCACCACGCUUCAUUUCAAGUAAAGUAUCGAUUCGUCGGUAUGAUUCUCCAUUAUAGCUCUUG**G**UAAACGGUAAGUUCAU
Rasp1-R1_Rep2 7301 UGUCACUUGGAUUGAGGCACUCACCACGCUUCAUUUCAAGUAAAGUAUCGAUUCGUCGGUAUGAUUCUCCAUUAUAGCUCUUG**G**UAAACGGUAAGUUCAU
Rasp1-R1_Rep3 7301 UGUCACUUGGAUUGAGGCACUCACCACGCUUCAUUUCAAGUAAAGUAUCGAUUCGUCGGUAUGAUUCUCCAUUAUAGCUCUUG**A**UAAACGGUAAGUUCAU

Rasp1-R1_2014 7401 UGAUCGCGUUAGAGUGUGGAAAAUAGUCUGAAACGAACUCAGUACCAGAGGUAGGACGCUAUUGUUCCAGGCGUUUCUUAUGGGCAUAAGCUGUAAACUU
Rasp1-R1_Rep1 7401 UGAUCGCGUUAGAGUGUGGAAAAUAGUCUGAAACGAACUCAGUACCAGAGGUAGGACGCUAUUGUUCCAGGCGUUUCUUAUGGGCAUAAGCUGUAAACUU
Rasp1-R1_Rep2 7401 UGAUCGCGUUAGAGUGUGGAAAAUAGUCUGAAACGAACUCAGUACCAGAGGUAGGACGCUAUUGUUCCAGGCGUUUCUUAUGGGCAUAAGCUGUAAACUU
Rasp1-R1_Rep3 7401 UGAUCGCGUUAGAGUGUGGAAAAUAGUCUGAAACGAACUCAGUACCAGAGGUAGGACGCUAUUGUUCCAGGCGUUUCUUAUGGGCAUAAGCUGUAAACUU

Rasp1-R1_2014 7501 GGUUUCGCAAGCCAUUCAGCACCUCCCUUUGCUUGUGUACUAUCUAGGGGCUCCCGGCCUUUCUUCCGGUACAAUACCUAGUGAAGCAAGCAAUUGCGUU
Rasp1-R1_Rep1 7501 GGUUUCGCAAGCCAUUCAGCACCUCCCUUUGCUUGUGUACUAUCUAGGGGCUCCCGGCCUUUCUUCCGGUACAAUACCUAGUGAAGCAAGCAAUUGCGUU
Rasp1-R1_Rep2 7501 GGUUUCGCAAGCCAUUCAGCACCUCCCUUUGCUUGUGUACUAUCUAGGGGCUCCCGGCCUUUCUUCCGGUACAAUACCUAGUGAAGCAAGCAAUUGCGUU
Rasp1-R1_Rep3 7501 GGUUUCGCAAGCCAUUCAGCACCUCCCUUUGCUUGUGUACUAUCUAGGGGCUCCCGGCCUUUCUUCCGGUACAAUACCUAGUGAAGCAAGCAAUUGCGUU

Rasp1-R1_2014 7601 GAGGGAUAAGAGUAGCAUGUUCCUGCUUAACGGAGGAAUAUGUCGUGUUUUCUACACGUUAGUGUUGCAUUGCUAUAAUGGCAAUGUAGUGCAGGAAUGG
Rasp1-R1_Rep1 7601 GAGGGAUAAGAGUAGCAUGUUCCUGCUUAACGGAGGAAUAUGUCGUGUUUUCUACACGUUAGUGUUGCAUUGCUAUAAUGGCAAUGUAGUGCAGGAAUGG
Rasp1-R1_Rep2 7601 GAGGGAUAAGAGUAGCAUGUUCCUGCUUAACGGAGGAAUAUGUCGUGUUUUCUACACGUUAGUGUUGCAUUGCUAUAAUGGCAAUGUAGUGCAGGAAUGG
Rasp1-R1_Rep3 7601 GAGGGAUAAGAGUAGCAUGUUCCUGCUUAACGGAGGAAUAUGUCGUGUUUUCUACACGUUAGUGUUGCAUUGCUAUAAUGGCAAUGUAGUGCAGGAAUGG

Rasp1-R1_2014 7701 UUCCCAGCCACUUUUUUCUGGGAUUCUAAUCGUACGUCACAAUUGUGUGUGUAUCGUUGACGGAGGAGUAGCGAUCCUCUACCACGCGAGUCUGGAAGUG
Rasp1-R1_Rep1 7701 UUCCCAGCCACUUUUUUCUGGGAUUCUAAUCGUACGUCACAAUUGUGUGUGUAUCGUUGACGGAGGAGUAGCGAUCCUCUACCACGCGAGUCUGGAAGUG
Rasp1-R1_Rep2 7701 UUCCCAGCCACUUUUUUCUGGGAUUCUAAUCGUACGUCACAAUUGUGUGUGUAUCGUUGACGGAGGAGUAGCGAUCCUCUACCACGCGAGUCUGGAAGUG
Rasp1-R1_Rep3 7701 UUCCCAGCCACUUUUUUCUGGGAUUCUAAUCGUACGUCACAAUUGUGUGUGUAUCGUUGACGGAGGAGUAGCGAUCCUCUACCACGCGAGUCUGGAAGUG

Rasp1-R1_2014 7801 AUUACCAGGGCCUAAGAUGGCCAGCACACGGUACGAUUAAAUUUAGUUGUAAUGUAGUGGUAUGUUAAGUUGAGACUAACUUACCCGUACGAGUCAAACU
Rasp1-R1_Rep1 7801 AUUACCAGGGCCUAAGAUGGCCAGCACACGGUACGAUUAAAUUUAGUUGUAAUGUAGUGGUAUGUUAAGUUGAGACUAACUUACCCGUACGAGUCAAACU
Rasp1-R1_Rep2 7801 AUUACCAGGGCCUAAGAUGGCCAGCACACGGUACGAUUAAAUUUAGUUGUAAUGUAGUGGUAUGUUAAGUUGAGACUAACUUACCCGUACGAGUCAAACU
Rasp1-R1_Rep3 7801 AUUACCAGGGCCUAAGAUGGCCAGCACACGGUACGAUUAAAUUUAGUUGUAAUGUAGUGGUAUGUUAAGUUGAGACUAACUUACCCGUACGAGUCAAACU

Rasp1-R1_2014 7901 UUAAGAUGGAUGUGUGUUCUGCCAUCUUGAGGGAAGUAGAUGUGGUUUUACCAAUCUGAGACGAGCCGUUAAUUCGGUGCUUUAAUACGUCAAUGAUAAU
Rasp1-R1_Rep1 7901 UUAAGAUGGAUGUGUGUUCUGCCAUCUUGAGGGAAGUAGAUGUGGUUUUACCAAUCUGAGACGAGCCGUUAAUUCGGUGCUUUAAUACGUCAAUGAUAAU
Rasp1-R1_Rep2 7901 UUAAGAUGGAUGUGUGUUCUGCCAUCUUGAGGGAAGUAGAUGUGGUUUUACCAAUCUGAGACGAGCCGUUAAUUCGGUGCUUUAAUACGUCAAUGAUAAU
Rasp1-R1_Rep3 7901 UUAAGAUGGAUGUGUGUUCUGCCAUCUUGAGGGAAGUAGAUGUGGUUUUACCAAUCUGAGACGAGCCGUUAAUUCGGUGCUUUAAUACGUCAAUGAUAAU

Rasp1-R1_2014 8001 ACUCGUGCAGUUGCAGCUGCACGAGUAUGUUGGUACACACAGUCUACUCGGAUACGGUCGAGUUGCCCUCACAACAGGGAUUACUCUCUCAAUCUUAACU
Rasp1-R1_Rep1 8001 ACUCGUGCAGUUGCAGCUGCACGAGUAUGUUGGUACACACAGUCUACUCGGAUACGGUCGAGUUGCCCUCACAACAGGGAUUACUCUCUCAAUCUUAACU
Rasp1-R1_Rep2 8001 ACUCGUGCAGUUGCAGCUGCACGAGUAUGUUGGUACACACAGUCUACUCGGAUACGGUCGAGUUGCCCUCACAACAGGGAUUACUCUCUCAAUCUUAACU
Rasp1-R1_Rep3 8001 ACUCGUGCAGUUGCAGCUGCACGAGUAUGUUGGUACACACAGUCUACUCGGAUACGGUCGAGUUGCCCUCACAACAGGGAUUACUCUCUCAAUCUUAACU

Rasp1-R1_2014 8101 ACUGCAAGGACGUUGUUUUCGCAGGGUUUUGUUGGUCCGCUUGUGUUUCAAAACGCUGCUUUGCAAUUUUCUUUUUUGUUUUAUUGCUUUCGUAGUGUCG
Rasp1-R1_Rep1 8101 ACUGCAAGGACGUUGUUUUCGCAGGGUUUUGUUGGUCCGCUUGUGUUUCAAAACGCUGCUUUGCAAUUUUCUUUUUUGUUUUAUUGCUUUCGUAGUGUCG
Rasp1-R1_Rep2 8101 ACUGCAAGGACGUUGUUUUCGCAGGGUUUUGUUGGUCCGCUUGUGUUUCAAAACGCUGCUUUGCAAUUUUCUUUUUUGUUUUAUUGCUUUCGUAGUGUCG
Rasp1-R1_Rep3 8101 ACUGCAAGGACGUUGUUUUCGCAGGGUUUUGUUGGUCCGCUUGUGUUUCAAAACGCUGCUUUGCAAUUUUCUUUUUUGUUUUAUUGCUUUCGUAGUGUCG

Rasp1-R1_2014 8201 AACUUUGUCCAAGUUCAUAAAAGC
Rasp1-R1_Rep1 8201 AACUUUGUCCAAGUUCAUAAAAGC
Rasp1-R1_Rep2 8201 AACUUUGUCCAAGUUCAUAAAAGC
Rasp1-R1_Rep3 8201 AACUUUGUCCAAGUUCAUAAAAGC
