# Supplementary material for: Transcriptomic changes associated with infection of Nicotiana benthamiana plants with tomato ringspot virus (genus Nepovirus) during the acute symptomatic stage and after symptom recovery
Source: PLoS One. 2025 Sep 2;20(9):e0328517. doi: 10.1371/journal.pone.0328517 (PMC12404439; doi:10.1371/journal.pone.0328517)
Supplement: S2 Text — (DOCX) [file pone.0328517.s005.docx]

**S2 Text. Alignment of the nucleotide sequence of RNA2 comparing the original 2014 sequence to that assembled from RNA seq data for each biological repeat (Rep1, Rep2, Rep3).** Differences in the nucleotide sequences are highlighted with red font.

Rasp1-R2_2014 1 UUGCGAAAAAUCUGGUGAUAUUCCAAGUUCUCUCGAUUUACACUUCCAUUGUGCCGUUUUGUUUUCUUUUCUUUUGAUGUCCUCCAUUUGUUUCGCCGGU
Rasp1-R2_Rep1 1 UUGCGAAAAAUCUGGUGAUAUUCCAAGUUCUCUCGAUUUACACUUCCAUUGUGCCGUUUUGUUUUCUUUUCUUUUGAUGUCCUCCAUUUGUUUCGCCGGU
Rasp1-R2_Rep2 1 UUGCGAAAAAUCUGGUGAUAUUCCAAGUUCUCUCGAUUUACACUUCCAUUGUGCCGUUUUGUUUUCUUUUCUUUUGAUGUCCUCCAUUUGUUUCGCCGGU
Rasp1-R2_Rep3 1 UUGCGAAAAAUCUGGUGAUAUUCCAAGUUCUCUCGAUUUACACUUCCAUUGUGCCGUUUUGUUUUCUUUUCUUUUGAUGUCCUCCAUUUGUUUCGCCGGU

Rasp1-R2_2014 101 GGCAACCACGCUAGGUUGCCAUCGAAAGCUGCUUUCCGUCGGGCUAUGUCCGAUGGGGAUCUGGACCGCGAGGGUCGCUUCCCUUGCGGGUGUCUAGCAC
Rasp1-R2_Rep1 101 GGCAACCACGCUAGGUUGCCAUCGAAAGCUGCUUUCCGUCGGGCUAUGUCCGAUGGGGAUCUGGACCGCGAGGGUCGCUUCCCUUGCGGGUGUCUAGCAC
Rasp1-R2_Rep2 101 GGCAACCACGCUAGGUUGCCAUCGAAAGCUGCUUUCCGUCGGGCUAUGUCCGAUGGGGAUCUGGACCGCGAGGGUCGCUUCCCUUGCGGGUGUCUAGCAC
Rasp1-R2_Rep3 101 GGCAACCACGCUAGGUUGCCAUCGAAAGCUGCUUUCCGUCGGGCUAUGUCCGAUGGGGAUCUGGACCGCGAGGGUCGCUUCCCUUGCGGGUGUCUAGCAC

Rasp1-R2_2014 201 AGUAUACUGUGCAAGCCCCCCCUCCUGCCAAGACACAGGAGACAGUCGUAGGCAGGUCCGCUGACCUCCAAAAGGGUAAUGUUGCUCCCCUUAAGAAGCA
Rasp1-R2_Rep1 201 AGUAUACUGUGCAAGCCCCCCCUCCUGCCAAGACACAGGAGACAGUCGUAGGCAGGUCCGCUGACCUCCAAAAGGGUAAUGUUGCUCCCCUUAAGAAGCA
Rasp1-R2_Rep2 201 AGUAUACUGUGCAAGCCCCCCCUCCUGCCAAGACACAGGAGACAGUCGUAGGCAGGUCCGCUGACCUCCAAAAGGGUAAUGUUGCUCCCCUUAAGAAGCA
Rasp1-R2_Rep3 201 AGUAUACUGUGCAAGCCCCCCCUCCUGCCAAGACACAGGAGACAGUCGUAGGCAGGUCCGCUGACCUCCAAAAGGGUAAUGUUGCUCCCCUUAAGAAGCA

Rasp1-R2_2014 301 ACGCUGCGAUGUUGUGGUCGCAGUCUCUGGACCUCCUCCUUUGGAGUUGGUCUACCCUGCCCGGGUAGGGCAGCAUAGGUUGGACCAACCUUCAAAAGGU
Rasp1-R2_Rep1 301 ACGCUGCGAUGUUGUGGUCGCAGUCUCUGGACCUCCUCCUUUGGAGUUGGUCUACCCUGCCCGGGUAGGGCAGCAUAGGUUGGACCAACCUUCAAAAGGU
Rasp1-R2_Rep2 301 ACGCUGCGAUGUUGUGGUCGCAGUCUCUGGACCUCCUCCUUUGGAGUUGGUCUACCCUGCCCGGGUAGGGCAGCAUAGGUUGGACCAACCUUCAAAAGGU
Rasp1-R2_Rep3 301 ACGCUGCGAUGUUGUGGUCGCAGUCUCUGGACCUCCUCCUUUGGAGUUGGUCUACCCUGCCCGGGUAGGGCAGCAUAGGUUGGACCAACCUUCAAAAGGU

Rasp1-R2_2014 401 CCCUUGGCAGUCCCCUCUGCCAAGCAAACCUCCACUGCAAUGGAGGUUGUUCUUUCUGUCCAGGAGGCGGCUAUCACUGCCCCCUGGCUUCUUCGCCCCU
Rasp1-R2_Rep1 401 CCCUUGGCAGUCCCCUCUGCCAAGCAAACCUCCACUGCAAUGGAGGUUGUUCUUUCUGUCCAGGAGGCGGCUAUCACUGCCCCCUGGCUUCUUCGCCCCU
Rasp1-R2_Rep2 401 CCCUUGGCAGUCCCCUCUGCCAAGCAAACCUCCACUGCAAUGGAGGUUGUUCUUUCUGUCCAGGAGGCGGCUAUCACUGCCCCCUGGCUUCUUCGCCCCU
Rasp1-R2_Rep3 401 CCCUUGGCAGUCCCCUCUGCCAAGCAAACCUCCACUGCAAUGGAGGUUGUUCUUUCUGUCCAGGAGGCGGCUAUCACUGCCCCCUGGCUUCUUCGCCCCU

Rasp1-R2_2014 501 GCAAGGGUGAAGCUUCCCCCCCCCCCCCCCUUACACAAAGGCAGCAAUUCGCUGCCCUAAAGAAGAGGCUGGCCGCCAAGGGCCAGCAAAUUAUCCGCGA
Rasp1-R2_Rep1 501 GCAAGGGUGAAGCUUCCCCCCCCCCCCCCCUUACACAAAGGCAGCAAUUCGCUGCCCUAAAGAAGAGGCUGGCCGCCAAGGGCCAGCAAAUUAUCCGCGA
Rasp1-R2_Rep2 501 GCAAGGGUGAAGCUUCCCCCCCCCCCCCCCUUACACAAAGGCAGCAAUUCGCUGCCCUAAAGAAGAGGCUGGCCGCCAAGGGCCAGCAAAUUAUCCGCGA
Rasp1-R2_Rep3 501 GCAAGGGUGAAGCUUCCCCCCCCCCCCCCCUUACACAAAGGCAGCAAUUCGCUGCCCUAAAGAAGAGGCUGGCCGCCAAGGGCCAGCAAAUUAUCCGCGA

Rasp1-R2_2014 601 GCACAUUCGUGCUCGCAAGGCGGCCAAAUAUGCCGCCAUCGCCAAAGCCAAAAAGGCUGCGGCUCUUGCUGCCGUUAAGGCAGCGCAGGAGGCUCCUCGC
Rasp1-R2_Rep1 601 GCACAUUCGUGCUCGCAAGGCGGCCAAAUAUGCCGCCAUCGCCAAAGCCAAAAAGGCUGCGGCUCUUGCUGCCGUUAAGGCAGCGCAGGAGGCUCCUCGC
Rasp1-R2_Rep2 601 GCACAUUCGUGCUCGCAAGGCGGCCAAAUAUGCCGCCAUCGCCAAAGCCAAAAAGGCUGCGGCUCUUGCUGCCGUUAAGGCAGCGCAGGAGGCUCCUCGC
Rasp1-R2_Rep3 601 GCACAUUCGUGCUCGCAAGGCGGCCAAAUAUGCCGCCAUCGCCAAAGCCAAAAAGGCUGCGGCUCUUGCUGCCGUUAAGGCAGCGCAGGAGGCUCCUCGC

Rasp1-R2_2014 701 CUUGCGGCCCAAAAGGCUGCCAUCAGCAAGAUCCUUAGGGAUCGAGCUGUUGCUGCUCUCCCCCUUCCUCCUCCUCCUUCUGCUGCCAGAUUGGCAGCUG
Rasp1-R2_Rep1 701 CUUGCGGCCCAAAAGGCUGCCAUCAGCAAGAUCCUUAGGGAUCGAGCUGUUGCUGCUCUCCCCCUUCCUCCUCCUCCUUCUGCUGCCAGAUUGGCAGCUG
Rasp1-R2_Rep2 701 CUUGCGGCCCAAAAGGCUGCCAUCAGCAAGAUCCUUAGGGAUCGAGCUGUUGCUGCUCUCCCCCUUCCUCCUCCUCCUUCUGCUGCCAGAUUGGCAGCUG
Rasp1-R2_Rep3 701 CUUGCGGCCCAAAAGGCUGCCAUCAGCAAGAUCCUUAGGGAUCGAGCUGUUGCUGCUCUCCCCCUUCCUCCUCCUCCUUCUGCUGCCAGAUUGGCAGCUG

Rasp1-R2_2014 801 AGGCCGAAUUGGCCUCAAAGGCCGAGUCUCUUCGGAGGCUCAAGGCCUUUCGCAAGUUCAGCAGGGUUCGCCCUGCUUUAAACACUUCUUUUCUUUCUCC
Rasp1-R2_Rep1 801 AGGCCGAAUUGGCCUCAAAGGCCGAGUCUCUUCGGAGGCUCAAGGCCUUUCGCAAGUUCAGCAGGGUUCGCCCUGCUUUAAACACUUCUUUUCUUUCUCC
Rasp1-R2_Rep2 801 AGGCCGAAUUGGCCUCAAAGGCCGAGUCUCUUCGGAGGCUCAAGGCCUUUCGCAAGUUCAGCAGGGUUCGCCCUGCUUUAAACACUUCUUUUCUUUCUCC
Rasp1-R2_Rep3 801 AGGCCGAAUUGGCCUCAAAGGCCGAGUCUCUUCGGAGGCUCAAGGCCUUUCGCAAGUUCAGCAGGGUUCGCCCUGCUUUAAACACUUCUUUUCUUUCUCC

Rasp1-R2_2014 901 UCCCCCUUCCCCUCCGGCUCGGUCUUCCGAGCUUUUGGCGGCUUUUAGUGCCGCCAUGAACAGGUCUCAGCCUGUUCAAGGGGGUUUUUCCCUUCCUGCC
Rasp1-R2_Rep1 901 UCCCCCUUCCCCUCCGGCUCGGUCUUCCGAGCUUUUGGCGGCUUUUAGUGCCGCCAUGAACAGGUCUCAGCCUGUUCAAGGGGGUUUUUCCCUUCCUGCC
Rasp1-R2_Rep2 901 UCCCCCUUCCCCUCCGGCUCGGUCUUCCGAGCUUUUGGCGGCUUUUAGUGCCGCCAUGAACAGGUCUCAGCCUGUUCAAGGGGGUUUUUCCCUUCCUGCC
Rasp1-R2_Rep3 901 UCCCCCUUCCCCUCCGGCUCGGUCUUCCGAGCUUUUGGCGGCUUUUAGUGCCGCCAUGAACAGGUCUCAGCCUGUUCAAGGGGGUUUUUCCCUUCCUGCC

Rasp1-R2_2014 1001 CGCAAGGGCGUUUAUGUCGCUCCCACCAUUCAGGGUGUGGUGCGUGCUGGGCUUCGUGCUCAGAAGGGCUUUUUAAAUGCCGUUUCUGCCGGCAUUGUGG
Rasp1-R2_Rep1 1001 CGCAAGGGCGUUUAUGUCGCUCCCACCAUUCAGGGUGUGGUGCGUGCUGGGCUUCGUGCUCAGAAGGGCUUUUUAAAUGCCGUUUCUGCCGGCAUUGUGG
Rasp1-R2_Rep2 1001 CGCAAGGGCGUUUAUGUCGCUCCCACCAUUCAGGGUGUGGUGCGUGCUGGGCUUCGUGCUCAGAAGGGCUUUUUAAAUGCCGUUUCUGCCGGCAUUGUGG
Rasp1-R2_Rep3 1001 CGCAAGGGCGUUUAUGUCGCUCCCACCAUUCAGGGUGUGGUGCGUGCUGGGCUUCGUGCUCAGAAGGGCUUUUUAAAUGCCGUUUCUGCCGGCAUUGUGG

Rasp1-R2_2014 1101 CUGGAGCUCGUAUUUUAAAGAGCAAAAGUCAAAAUUGGUUUAGAAAGAGCAUGGGCAUUGCCCAUGAGUAUGUGGAGGGUUCUCUGGCCAGCACCAUUUU
Rasp1-R2_Rep1 1101 CUGGAGCUCGUAUUUUAAAGAGCAAAAGUCAAAAUUGGUUUAGAAAGAGCAUGGGCAUUGCCCAUGAGUAUGUGGAGGGUUCUCUGGCCAGCACCAUUUU
Rasp1-R2_Rep2 1101 CUGGAGCUCGUAUUUUAAAGAGCAAAAGUCAAAAUUGGUUUAGAAAGAGCAUGGGCAUUGCCCAUGAGUAUGUGGAGGGUUCUCUGGCCAGCACCAUUUU
Rasp1-R2_Rep3 1101 CUGGAGCUCGUAUUUUAAAGAGCAAAAGUCAAAAUUGGUUUAGAAAGAGCAUGGGCAUUGCCCAUGAGUAUGUGGAGGGUUCUCUGGCCAGCACCAUUUU

Rasp1-R2_2014 1201 AGAGUGUGCUGGUCCAGUUGUGCAAAGUAAGGAGAGCUGCGCUGUUGUUGCAGUUCCCCCAUCUGAGGAGCUCAUUUCUUGGGUUCCCUCUUGCACUGAG
Rasp1-R2_Rep1 1201 AGAGUGUGCUGGUCCAGUUGUGCAAAGUAAGGAGAGCUGCGCUGUUGUUGCAGUUCCCCCAUCUGAGGAGCUCAUUUCUUGGGUUCCCUCUUGCACUGAG
Rasp1-R2_Rep2 1201 AGAGUGUGCUGGUCCAGUUGUGCAAAGUAAGGAGAGCUGCGCUGUUGUUGCAGUUCCCCCAUCUGAGGAGCUCAUUUCUUGGGUUCCCUCUUGCACUGAG
Rasp1-R2_Rep3 1201 AGAGUGUGCUGGUCCAGUUGUGCAAAGUAAGGAGAGCUGCGCUGUUGUUGCAGUUCCCCCAUCUGAGGAGCUCAUUUCUUGGGUUCCCUCUUGCACUGAG

Rasp1-R2_2014 1301 UAUGCGAGCAAUUUUCCUAGGCUCACAUGUUCUAUUUUUACUGAAUGGCAAAAACCGCGCAAGCAGUCCAUUGUCGUUUUCAAUCCUUUCCGCAAGCUCA
Rasp1-R2_Rep1 1301 UAUGCGAGCAAUUUUCCUAGGCUCACAUGUUCUAUUUUUACUGAAUGGCAAAAACCGCGCAAGCAGUCCAUUGUCGUUUUCAAUCCUUUCCGCAAGCUCA
Rasp1-R2_Rep2 1301 UAUGCGAGCAAUUUUCCUAGGCUCACAUGUUCUAUUUUUACUGAAUGGCAAAAACCGCGCAAGCAGUCCAUUGUCGUUUUCAAUCCUUUCCGCAAGCUCA
Rasp1-R2_Rep3 1301 UAUGCGAGCAAUUUUCCUAGGCUCACAUGUUCUAUUUUUACUGAAUGGCAAAAACCGCGCAAGCAGUCCAUUGUCGUUUUCAAUCCUUUCCGCAAGCUCA

Rasp1-R2_2014 1401 UCGAUUGUGCUUUAAUCAUGAGUGGCGUUAGCUUAAUCGCGAGCGUCCUUUUACGUGAGGUUGCGGAAAAUCUUGUAGUGCGACAGGUAGUUCACCCAAG
Rasp1-R2_Rep1 1401 UCGAUUGUGCUUUAAUCAUGAGUGGCGUUAGCUUAAUCGCGAGCGUCCUUUUACGUGAGGUUGCGGAAAAUCUUGUAGUGCGACAGGUAGUUCACCCAAG
Rasp1-R2_Rep2 1401 UCGAUUGUGCUUUAAUCAUGAGUGGCGUUAGCUUAAUCGCGAGCGUCCUUUUACGUGAGGUUGCGGAAAAUCUUGUAGUGCGACAGGUAGUUCACCCAAG
Rasp1-R2_Rep3 1401 UCGAUUGUGCUUUAAUCAUGAGUGGCGUUAGCUUAAUCGCGAGCGUCCUUUUACGUGAGGUUGCGGAAAAUCUUGUAGUGCGACAGGUAGUUCACCCAAG

Rasp1-R2_2014 1501 GGAAAUGCUACCUUGUGCAGUUUUUGUCUCGGAGAAAUCCUUAGUCCCACUGGACGAAUGGAGUUUCUAUCUCCGCAAAUAUUUGUCACCACCUCCCUAU
Rasp1-R2_Rep1 1501 GGAAAUGCUACCUUGUGCAGUUUUUGUCUCGGAGAAAUCCUUAGUCCCACUGGACGAAUGGAGUUUCUAUCUCCGCAAAUAUUUGUCACCACCUCCCUAU
Rasp1-R2_Rep2 1501 GGAAAUGCUACCUUGUGCAGUUUUUGUCUCGGAGAAAUCCUUAGUCCCACUGGACGAAUGGAGUUUCUAUCUCCGCAAAUAUUUGUCACCACCUCCCUAU
Rasp1-R2_Rep3 1501 GGAAAUGCUACCUUGUGCAGUUUUUGUCUCGGAGAAAUCCUUAGUCCCACUGGACGAAUGGAGUUUCUAUCUCCGCAAAUAUUUGUCACCACCUCCCUAU

Rasp1-R2_2014 1601 CCUUUUGGUAGGGAAAGUUUCUAUUUUCAAGCUAGGCCCCGUUUUAUUGGACCUAUGCUUUCUAUGGUUAGGGCUGUACCACGAAUUGUACAACAGCCCA
Rasp1-R2_Rep1 1601 CCUUUUGGUAGGGAAAGUUUCUAUUUUCAAGCUAGGCCCCGUUUUAUUGGACCUAUGCUUUCUAUGGUUAGGGCUGUACCACGAAUUGUACAACAGCCCA
Rasp1-R2_Rep2 1601 CCUUUUGGUAGGGAAAGUUUCUAUUUUCAAGCUAGGCCCCGUUUUAUUGGACCUAUGCUUUCUAUGGUUAGGGCUGUACCACGAAUUGUACAACAGCCCA
Rasp1-R2_Rep3 1601 CCUUUUGGUAGGGAAAGUUUCUAUUUUCAAGCUAGGCCCCGUUUUAUUGGACCUAUGCUUUCUAUGGUUAGGGCUGUACCACGAAUUGUACAACAGCCCA

Synonymous mutation - Cys (UGC_1792_) -> Cys (UGU_1792_)

Rasp1-R2_2014 1701 CCAUGACGGAGGAACUCGAAUUUGAAGUUCCUUCCUCAUGGUCUUCUCCUUUACCUCUAUUUGCGAAUUUUAAAGUAAAUAGGGGCGCAUG**C**UUUUUGCA
Rasp1-R2_Rep1 1701 CCAUGACGGAGGAACUCGAAUUUGAAGUUCCUUCCUCAUGGUCUUCUCCUUUACCUCUAUUUGCGAAUUUUAAAGUAAAUAGGGGCGCAUG**C**UUUUUGCA
Rasp1-R2_Rep2 1701 CCAUGACGGAGGAACUCGAAUUUGAAGUUCCUUCCUCAUGGUCUUCUCCUUUACCUCUAUUUGCGAAUUUUAAAGUAAAUAGGGGCGCAUG**C**UUUUUGCA
Rasp1-R2_Rep3 1701 CCAUGACGGAGGAACUCGAAUUUGAAGUUCCUUCCUCAUGGUCUUCUCCUUUACCUCUAUUUGCGAAUUUUAAAGUAAAUAGGGGCGCAUG**U**UUUUUGCA

Rasp1-R2_2014 1801 AGUCCUGCCUCAAAGGGUUGUUUUACCUGAUGAAUGCAUGGAUUUGCUUUCUCUUUUUGAGGAUCAAUUGCCAGAGGGGCCUUUGCCCCCCUUUAGUUGG
Rasp1-R2_Rep1 1801 AGUCCUGCCUCAAAGGGUUGUUUUACCUGAUGAAUGCAUGGAUUUGCUUUCUCUUUUUGAGGAUCAAUUGCCAGAGGGGCCUUUGCCCCCCUUUAGUUGG
Rasp1-R2_Rep2 1801 AGUCCUGCCUCAAAGGGUUGUUUUACCUGAUGAAUGCAUGGAUUUGCUUUCUCUUUUUGAGGAUCAAUUGCCAGAGGGGCCUUUGCCCCCCUUUAGUUGG
Rasp1-R2_Rep3 1801 AGUCCUGCCUCAAAGGGUUGUUUUACCUGAUGAAUGCAUGGAUUUGCUUUCUCUUUUUGAGGAUCAAUUGCCAGAGGGGCCUUUGCCCCCCUUUAGUUGG

Rasp1-R2_2014 1901 UCUUCUCCUUUACCUCUAUUUGCGAAUUUUAAAGUAAAUAGGGGCGCAUGUUUUUUGCAAGUCCUGCCUCAAAGGGUUGUUUUACCUGAUGAAUGCAUGG
Rasp1-R2_Rep1 1901 UCUUCUCCUUUACCUCUAUUUGCGAAUUUUAAAGUAAAUAGGGGCGCAUGUUUUUUGCAAGUCCUGCCUCAAAGGGUUGUUUUACCUGAUGAAUGCAUGG
Rasp1-R2_Rep2 1901 UCUUCUCCUUUACCUCUAUUUGCGAAUUUUAAAGUAAAUAGGGGCGCAUGUUUUUUGCAAGUCCUGCCUCAAAGGGUUGUUUUACCUGAUGAAUGCAUGG
Rasp1-R2_Rep3 1901 UCUUCUCCUUUACCUCUAUUUGCGAAUUUUAAAGUAAAUAGGGGCGCAUGUUUUUUGCAAGUCCUGCCUCAAAGGGUUGUUUUACCUGAUGAAUGCAUGG

Rasp1-R2_2014 2001 AUUUGCUUUCUCUUUUUGAGGAUCAAUUGCCAGAGGGGCCUUUGCCCCCCUUUAGUUGGUCUUCUCCUUUACCUCUAUUUGCGAAUUUUAAAGUAAAUAG
Rasp1-R2_Rep1 2001 AUUUGCUUUCUCUUUUUGAGGAUCAAUUGCCAGAGGGGCCUUUGCCCCCCUUUAGUUGGUCUUCUCCUUUACCUCUAUUUGCGAAUUUUAAAGUAAAUAG
Rasp1-R2_Rep2 2001 AUUUGCUUUCUCUUUUUGAGGAUCAAUUGCCAGAGGGGCCUUUGCCCCCCUUUAGUUGGUCUUCUCCUUUACCUCUAUUUGCGAAUUUUAAAGUAAAUAG
Rasp1-R2_Rep3 2001 AUUUGCUUUCUCUUUUUGAGGAUCAAUUGCCAGAGGGGCCUUUGCCCCCCUUUAGUUGGUCUUCUCCUUUACCUCUAUUUGCGAAUUUUAAAGUAAAUAG

Rasp1-R2_2014 2101 GGGCGCAUGUUUUUUGCAAGUCCUGCCUCAAAGGGUUGUUUUACCUGAUGAAUGCAUGGAUUUGCUUUCUCUUUUUGAGGAUCAAUUGCCAGAGGGGCCU
Rasp1-R2_Rep1 2101 GGGCGCAUGUUUUUUGCAAGUCCUGCCUCAAAGGGUUGUUUUACCUGAUGAAUGCAUGGAUUUGCUUUCUCUUUUUGAGGAUCAAUUGCCAGAGGGGCCU
Rasp1-R2_Rep2 2101 GGGCGCAUGUUUUUUGCAAGUCCUGCCUCAAAGGGUUGUUUUACCUGAUGAAUGCAUGGAUUUGCUUUCUCUUUUUGAGGAUCAAUUGCCAGAGGGGCCU
Rasp1-R2_Rep3 2101 GGGCGCAUGUUUUUUGCAAGUCCUGCCUCAAAGGGUUGUUUUACCUGAUGAAUGCAUGGAUUUGCUUUCUCUUUUUGAGGAUCAAUUGCCAGAGGGGCCU

Synonymous mutation - Cys (UGU_2269_) -> Cys (UGC_2269_)

Rasp1-R2_2014 2201 UUGCCCCCCUUUAGUUGGUCUUCACCUUUACCUCUGUUCACGAGUUUCAAAGUGAAUAGAGGCGCAUG**U**UUUUUGCAGGUUUUGCCUGCACGCAGGGUCG
Rasp1-R2_Rep1 2201 UUGCCCCCCUUUAGUUGGUCUUCACCUUUACCUCUGUUCACGAGUUUCAAAGUGAAUAGAGGCGCAUG**C**UUUUUGCAGGUUUUGCCUGCACGCAGGGUCG
Rasp1-R2_Rep2 2201 UUGCCCCCCUUUAGUUGGUCUUCACCUUUACCUCUGUUCACGAGUUUCAAAGUGAAUAGAGGCGCAUG**C**UUUUUGCAGGUUUUGCCUGCACGCAGGGUCG
Rasp1-R2_Rep3 2201 UUGCCCCCCUUUAGUUGGUCUUCACCUUUACCUCUGUUCACGAGUUUCAAAGUGAAUAGAGGCGCAUG**C**UUUUUGCAGGUUUUGCCUGCACGCAGGGUCG

Rasp1-R2_2014 2301 UUCUUGAUGAGUUUAUGGACGUCUUGCCCUUUUUGCUUUCUCCACCGGUAUUGCACCAGGAAGAGGAGCCAGAAAUGGCCCCUGCUGUGUUGGAAGCAGC
Rasp1-R2_Rep1 2301 UUCUUGAUGAGUUUAUGGACGUCUUGCCCUUUUUGCUUUCUCCACCGGUAUUGCACCAGGAAGAGGAGCCAGAAAUGGCCCCUGCUGUGUUGGAAGCAGC
Rasp1-R2_Rep2 2301 UUCUUGAUGAGUUUAUGGACGUCUUGCCCUUUUUGCUUUCUCCACCGGUAUUGCACCAGGAAGAGGAGCCAGAAAUGGCCCCUGCUGUGUUGGAAGCAGC
Rasp1-R2_Rep3 2301 UUCUUGAUGAGUUUAUGGACGUCUUGCCCUUUUUGCUUUCUCCACCGGUAUUGCACCAGGAAGAGGAGCCAGAAAUGGCCCCUGCUGUGUUGGAAGCAGC

Rasp1-R2_2014 2401 AACAACUCCACUGGUAUUGCACCAGGAAGAGGAGCCAGAAAUGGCCCCUGCUGUGUUGGAAGCAGUAACAACUCCACUGGUAUUGCACCAGGAAGAGGAA
Rasp1-R2_Rep1 2401 AACAACUCCACUGGUAUUGCACCAGGAAGAGGAGCCAGAAAUGGCCCCUGCUGUGUUGGAAGCAGUAACAACUCCACUGGUAUUGCACCAGGAAGAGGAA
Rasp1-R2_Rep2 2401 AACAACUCCACUGGUAUUGCACCAGGAAGAGGAGCCAGAAAUGGCCCCUGCUGUGUUGGAAGCAGUAACAACUCCACUGGUAUUGCACCAGGAAGAGGAA
Rasp1-R2_Rep3 2401 AACAACUCCACUGGUAUUGCACCAGGAAGAGGAGCCAGAAAUGGCCCCUGCUGUGUUGGAAGCAGUAACAACUCCACUGGUAUUGCACCAGGAAGAGGAA

Rasp1-R2_2014 2501 UCAAGAAUGGUUCCUGCUGUGUUGGAAGCAGCAAAUUCUAUUGAUGAUGUCACCGAAGCCUUCUUUGAUGACUUGGAAUGUGAGUCUUUCUAUGACUCAU
Rasp1-R2_Rep1 2501 UCAAGAAUGGUUCCUGCUGUGUUGGAAGCAGCAAAUUCUAUUGAUGAUGUCACCGAAGCCUUCUUUGAUGACUUGGAAUGUGAGUCUUUCUAUGACUCAU
Rasp1-R2_Rep2 2501 UCAAGAAUGGUUCCUGCUGUGUUGGAAGCAGCAAAUUCUAUUGAUGAUGUCACCGAAGCCUUCUUUGAUGACUUGGAAUGUGAGUCUUUCUAUGACUCAU
Rasp1-R2_Rep3 2501 UCAAGAAUGGUUCCUGCUGUGUUGGAAGCAGCAAAUUCUAUUGAUGAUGUCACCGAAGCCUUCUUUGAUGACUUGGAAUGUGAGUCUUUCUAUGACUCAU

Rasp1-R2_2014 2601 AUUCUUGUGAAGAAGAAGCUGAGUGGGCUGAAGUGCCAAGGUGUAAGACUAUGUCUGAACUUUACGCUUCUCUCACUUUUGUUGGUGACGCCGAGGGGCU
Rasp1-R2_Rep1 2601 AUUCUUGUGAAGAAGAAGCUGAGUGGGCUGAAGUGCCAAGGUGUAAGACUAUGUCUGAACUUUACGCUUCUCUCACUUUUGUUGGUGACGCCGAGGGGCU
Rasp1-R2_Rep2 2601 AUUCUUGUGAAGAAGAAGCUGAGUGGGCUGAAGUGCCAAGGUGUAAGACUAUGUCUGAACUUUACGCUUCUCUCACUUUUGUUGGUGACGCCGAGGGGCU
Rasp1-R2_Rep3 2601 AUUCUUGUGAAGAAGAAGCUGAGUGGGCUGAAGUGCCAAGGUGUAAGACUAUGUCUGAACUUUACGCUUCUCUCACUUUUGUUGGUGACGCCGAGGGGCU

Rasp1-R2_2014 2701 ACGUAAGUCUCACAUGACUUAUUUCCGCCGCCUUGUGGCUUACUUUCGGUCCUUCGAAGAGCCGCUUUACUCUUCACGCGCCUUUUAUAGCGUGAAGGUG
Rasp1-R2_Rep1 2701 ACGUAAGUCUCACAUGACUUAUUUCCGCCGCCUUGUGGCUUACUUUCGGUCCUUCGAAGAGCCGCUUUACUCUUCACGCGCCUUUUAUAGCGUGAAGGUG
Rasp1-R2_Rep2 2701 ACGUAAGUCUCACAUGACUUAUUUCCGCCGCCUUGUGGCUUACUUUCGGUCCUUCGAAGAGCCGCUUUACUCUUCACGCGCCUUUUAUAGCGUGAAGGUG
Rasp1-R2_Rep3 2701 ACGUAAGUCUCACAUGACUUAUUUCCGCCGCCUUGUGGCUUACUUUCGGUCCUUCGAAGAGCCGCUUUACUCUUCACGCGCCUUUUAUAGCGUGAAGGUG

Rasp1-R2_2014 2801 AAGCCAGUUUAUCGUCCCAAAAAAUUUGAGGGACAUAUCGAUUGUACCUGCCUCGAUGGCAAUAUGGGCGAGUGGGAAUGGCGCGAAAGCGUCGACGCCA
Rasp1-R2_Rep1 2801 AAGCCAGUUUAUCGUCCCAAAAAAUUUGAGGGACAUAUCGAUUGUACCUGCCUCGAUGGCAAUAUGGGCGAGUGGGAAUGGCGCGAAAGCGUCGACGCCA
Rasp1-R2_Rep2 2801 AAGCCAGUUUAUCGUCCCAAAAAAUUUGAGGGACAUAUCGAUUGUACCUGCCUCGAUGGCAAUAUGGGCGAGUGGGAAUGGCGCGAAAGCGUCGACGCCA
Rasp1-R2_Rep3 2801 AAGCCAGUUUAUCGUCCCAAAAAAUUUGAGGGACAUAUCGAUUGUACCUGCCUCGAUGGCAAUAUGGGCGAGUGGGAAUGGCGCGAAAGCGUCGACGCCA

Missense mutation - Gly (GG_2994_U) -> Asp (GA_2994_U)

Rasp1-R2_2014 2901 UGUGGCGUUGCCCAGGGCGCUUGCUCAAUGCAAAGCGCACAUAUACUCGCGAUGAUUGGGAGCGUGUGCAAUAUUUACGCAUAAGCUCCAAUG**G**UGGUAG
Rasp1-R2_Rep1 2901 UGUGGCGUUGCCCAGGGCGCUUGCUCAAUGCAAAGCGCACAUAUACUCGCGAUGAUUGGGAGCGUGUGCAAUAUUUACGCAUAAGCUCCAAUG**A**UGGUAG
Rasp1-R2_Rep2 2901 UGUGGCGUUGCCCAGGGCGCUUGCUCAAUGCAAAGCGCACAUAUACUCGCGAUGAUUGGGAGCGUGUGCAAUAUUUACGCAUAAGCUCCAAUG**A**UGGUAG
Rasp1-R2_Rep3 2901 UGUGGCGUUGCCCAGGGCGCUUGCUCAAUGCAAAGCGCACAUAUACUCGCGAUGAUUGGGAGCGUGUGCAAUAUUUACGCAUAAGCUCCAAUG**A**UGGUAG

Rasp1-R2_2014 3001 AUACCGCCAAAAUUGGCGCAUUUUAAACCUCGAGGAGAUGGAUCUCUCUUUGCAUGAAUAUCCAGAGAUUCCAUCUGCCCCAGUACAGUCCUCUCUUUUU
Rasp1-R2_Rep1 3001 AUACCGCCAAAAUUGGCGCAUUUUAAACCUCGAGGAGAUGGAUCUCUCUUUGCAUGAAUAUCCAGAGAUUCCAUCUGCCCCAGUACAGUCCUCUCUUUUU
Rasp1-R2_Rep2 3001 AUACCGCCAAAAUUGGCGCAUUUUAAACCUCGAGGAGAUGGAUCUCUCUUUGCAUGAAUAUCCAGAGAUUCCAUCUGCCCCAGUACAGUCCUCUCUUUUU
Rasp1-R2_Rep3 3001 AUACCGCCAAAAUUGGCGCAUUUUAAACCUCGAGGAGAUGGAUCUCUCUUUGCAUGAAUAUCCAGAGAUUCCAUCUGCCCCAGUACAGUCCUCUCUUUUU

Rasp1-R2_2014 3101 UCGAGGGUUGUCAAUAGGGGAGCCACCUUGGCAAGUAAUAUCCCCUUUGUCACUCGUUCUAACUGCCAGUCUUCUUUAGGAACUCCUGGUUCAAAUGUAC
Rasp1-R2_Rep1 3101 UCGAGGGUUGUCAAUAGGGGAGCCACCUUGGCAAGUAAUAUCCCCUUUGUCACUCGUUCUAACUGCCAGUCUUCUUUAGGAACUCCUGGUUCAAAUGUAC
Rasp1-R2_Rep2 3101 UCGAGGGUUGUCAAUAGGGGAGCCACCUUGGCAAGUAAUAUCCCCUUUGUCACUCGUUCUAACUGCCAGUCUUCUUUAGGAACUCCUGGUUCAAAUGUAC
Rasp1-R2_Rep3 3101 UCGAGGGUUGUCAAUAGGGGAGCCACCUUGGCAAGUAAUAUCCCCUUUGUCACUCGUUCUAACUGCCAGUCUUCUUUAGGAACUCCUGGUUCAAAUGUAC

Rasp1-R2_2014 3201 AUACUAUACACCAGGAAGCCCCUGCUACAUUGCGAGCUCCACCUUUUACAGGAGCGCGUAAUGUAGUGGGAUCCUCUGAUGCGGGUGCAAAUGCUGCCCC
Rasp1-R2_Rep1 3201 AUACUAUACACCAGGAAGCCCCUGCUACAUUGCGAGCUCCACCUUUUACAGGAGCGCGUAAUGUAGUGGGAUCCUCUGAUGCGGGUGCAAAUGCUGCCCC
Rasp1-R2_Rep2 3201 AUACUAUACACCAGGAAGCCCCUGCUACAUUGCGAGCUCCACCUUUUACAGGAGCGCGUAAUGUAGUGGGAUCCUCUGAUGCGGGUGCAAAUGCUGCCCC
Rasp1-R2_Rep3 3201 AUACUAUACACCAGGAAGCCCCUGCUACAUUGCGAGCUCCACCUUUUACAGGAGCGCGUAAUGUAGUGGGAUCCUCUGAUGCGGGUGCAAAUGCUGCCCC

Rasp1-R2_2014 3301 GUACCGCUCAGAAGCGCGCAAGCGCUGGCUGAGCCGUAAACAAGAGGAUUCCCAAGAAGAUAAUAUUAAGAAAUAUGCCGAUAAGCACGGCAUUUCCUUU
Rasp1-R2_Rep1 3301 GUACCGCUCAGAAGCGCGCAAGCGCUGGCUGAGCCGUAAACAAGAGGAUUCCCAAGAAGAUAAUAUUAAGAAAUAUGCCGAUAAGCACGGCAUUUCCUUU
Rasp1-R2_Rep2 3301 GUACCGCUCAGAAGCGCGCAAGCGCUGGCUGAGCCGUAAACAAGAGGAUUCCCAAGAAGAUAAUAUUAAGAAAUAUGCCGAUAAGCACGGCAUUUCCUUU
Rasp1-R2_Rep3 3301 GUACCGCUCAGAAGCGCGCAAGCGCUGGCUGAGCCGUAAACAAGAGGAUUCCCAAGAAGAUAAUAUUAAGAAAUAUGCCGAUAAGCACGGCAUUUCCUUU

Rasp1-R2_2014 3401 GAAGAGGCUAGGGCUGUUUACAAGGCCCCAAAGGAAGGAGUGCCCACCCAGCGCUCCAUUCUGCCUGAUGUCAGAGAUGCUUAUUCCGCCCGUUCUACUG
Rasp1-R2_Rep1 3401 GAAGAGGCUAGGGCUGUUUACAAGGCCCCAAAGGAAGGAGUGCCCACCCAGCGCUCCAUUCUGCCUGAUGUCAGAGAUGCUUAUUCCGCCCGUUCUACUG
Rasp1-R2_Rep2 3401 GAAGAGGCUAGGGCUGUUUACAAGGCCCCAAAGGAAGGAGUGCCCACCCAGCGCUCCAUUCUGCCUGAUGUCAGAGAUGCUUAUUCCGCCCGUUCUACUG
Rasp1-R2_Rep3 3401 GAAGAGGCUAGGGCUGUUUACAAGGCCCCAAAGGAAGGAGUGCCCACCCAGCGCUCCAUUCUGCCUGAUGUCAGAGAUGCUUAUUCCGCCCGUUCUACUG

Rasp1-R2_2014 3501 GCGCUCGGGUUCGGUCCCUCUUCGGAGGAUCCCCUACCACGCGCGCACAGAGGACGGAAGAUUUUGUGUUAACGAGCCCAUCUGCGGGGGAUGCAAGCUC
Rasp1-R2_Rep1 3501 GCGCUCGGGUUCGGUCCCUCUUCGGAGGAUCCCCUACCACGCGCGCACAGAGGACGGAAGAUUUUGUGUUAACGAGCCCAUCUGCGGGGGAUGCAAGCUC
Rasp1-R2_Rep2 3501 GCGCUCGGGUUCGGUCCCUCUUCGGAGGAUCCCCUACCACGCGCGCACAGAGGACGGAAGAUUUUGUGUUAACGAGCCCAUCUGCGGGGGAUGCAAGCUC
Rasp1-R2_Rep3 3501 GCGCUCGGGUUCGGUCCCUCUUCGGAGGAUCCCCUACCACGCGCGCACAGAGGACGGAAGAUUUUGUGUUAACGAGCCCAUCUGCGGGGGAUGCAAGCUC

Rasp1-R2_2014 3601 GUUUAGCUUUUAUUUUAAUCCUGUUUCUGAACAAGAGAUGGCUGAGCAAGAGCGUGGUGGUAAUACUAUGCUGUCUCUUGAUGCGGUUGAGGUCGUUAUU
Rasp1-R2_Rep1 3601 GUUUAGCUUUUAUUUUAAUCCUGUUUCUGAACAAGAGAUGGCUGAGCAAGAGCGUGGUGGUAAUACUAUGCUGUCUCUUGAUGCGGUUGAGGUCGUUAUU
Rasp1-R2_Rep2 3601 GUUUAGCUUUUAUUUUAAUCCUGUUUCUGAACAAGAGAUGGCUGAGCAAGAGCGUGGUGGUAAUACUAUGCUGUCUCUUGAUGCGGUUGAGGUCGUUAUU
Rasp1-R2_Rep3 3601 GUUUAGCUUUUAUUUUAAUCCUGUUUCUGAACAAGAGAUGGCUGAGCAAGAGCGUGGUGGUAAUACUAUGCUGUCUCUUGAUGCGGUUGAGGUCGUUAUU

Rasp1-R2_2014 3701 GACCCAGUUGGCAUGCCUGGUGAUGACACUGAUUUGACUGUUAUGGUCUUGUGGUGUCAAAAUUCAGAUGAUCAGCGUGCUCUGAUCGGGGCCAUGUCUA
Rasp1-R2_Rep1 3701 GACCCAGUUGGCAUGCCUGGUGAUGACACUGAUUUGACUGUUAUGGUCUUGUGGUGUCAAAAUUCAGAUGAUCAGCGUGCUCUGAUCGGGGCCAUGUCUA
Rasp1-R2_Rep2 3701 GACCCAGUUGGCAUGCCUGGUGAUGACACUGAUUUGACUGUUAUGGUCUUGUGGUGUCAAAAUUCAGAUGAUCAGCGUGCUCUGAUCGGGGCCAUGUCUA
Rasp1-R2_Rep3 3701 GACCCAGUUGGCAUGCCUGGUGAUGACACUGAUUUGACUGUUAUGGUCUUGUGGUGUCAAAAUUCAGAUGAUCAGCGUGCUCUGAUCGGGGCCAUGUCUA

Rasp1-R2_2014 3801 CUUUUGUGGGCAAUGGCCUGGCCAGAGCCGUUUUCUAUCCCGGGCUUAAAUUAUUAUAUGCCAAUUGUAGAGUGCGAGAUGGCCGAGUUUUAAAGGUCAU
Rasp1-R2_Rep1 3801 CUUUUGUGGGCAAUGGCCUGGCCAGAGCCGUUUUCUAUCCCGGGCUUAAAUUAUUAUAUGCCAAUUGUAGAGUGCGAGAUGGCCGAGUUUUAAAGGUCAU
Rasp1-R2_Rep2 3801 CUUUUGUGGGCAAUGGCCUGGCCAGAGCCGUUUUCUAUCCCGGGCUUAAAUUAUUAUAUGCCAAUUGUAGAGUGCGAGAUGGCCGAGUUUUAAAGGUCAU
Rasp1-R2_Rep3 3801 CUUUUGUGGGCAAUGGCCUGGCCAGAGCCGUUUUCUAUCCCGGGCUUAAAUUAUUAUAUGCCAAUUGUAGAGUGCGAGAUGGCCGAGUUUUAAAGGUCAU

Rasp1-R2_2014 3901 UGUGAGCAGCACGAAUUCAACGCUCACGCAUGGUUUGCCCCAGGCUCAAGUCUCCAUUGGGACUUUGCGCCAGCAUUUGGGGCCAGGUCAUGAUCGCACU
Rasp1-R2_Rep1 3901 UGUGAGCAGCACGAAUUCAACGCUCACGCAUGGUUUGCCCCAGGCUCAAGUCUCCAUUGGGACUUUGCGCCAGCAUUUGGGGCCAGGUCAUGAUCGCACU
Rasp1-R2_Rep2 3901 UGUGAGCAGCACGAAUUCAACGCUCACGCAUGGUUUGCCCCAGGCUCAAGUCUCCAUUGGGACUUUGCGCCAGCAUUUGGGGCCAGGUCAUGAUCGCACU
Rasp1-R2_Rep3 3901 UGUGAGCAGCACGAAUUCAACGCUCACGCAUGGUUUGCCCCAGGCUCAAGUCUCCAUUGGGACUUUGCGCCAGCAUUUGGGGCCAGGUCAUGAUCGCACU

Rasp1-R2_2014 4001 AUCUCUGGUGCCCUGUACGCUUCCCAACAACAGGGUUUCAAUAUACGCGCCACGGAACAAGGUGGUGCUGUAACAUUUGCCCCCCAAGGGGGCCAUGUUG
Rasp1-R2_Rep1 4001 AUCUCUGGUGCCCUGUACGCUUCCCAACAACAGGGUUUCAAUAUACGCGCCACGGAACAAGGUGGUGCUGUAACAUUUGCCCCCCAAGGGGGCCAUGUUG
Rasp1-R2_Rep2 4001 AUCUCUGGUGCCCUGUACGCUUCCCAACAACAGGGUUUCAAUAUACGCGCCACGGAACAAGGUGGUGCUGUAACAUUUGCCCCCCAAGGGGGCCAUGUUG
Rasp1-R2_Rep3 4001 AUCUCUGGUGCCCUGUACGCUUCCCAACAACAGGGUUUCAAUAUACGCGCCACGGAACAAGGUGGUGCUGUAACAUUUGCCCCCCAAGGGGGCCAUGUUG

Rasp1-R2_2014 4101 AGGGUAUCCCCAGCGCCAAUGUACAGAUGGGCGCCGGGGAGCAUUUAAUUCAAGCGGGUCCCAUGCAGUGGCGCUUGCAGAGGUCGCAAUCUUCUCGAUU
Rasp1-R2_Rep1 4101 AGGGUAUCCCCAGCGCCAAUGUACAGAUGGGCGCCGGGGAGCAUUUAAUUCAAGCGGGUCCCAUGCAGUGGCGCUUGCAGAGGUCGCAAUCUUCUCGAUU
Rasp1-R2_Rep2 4101 AGGGUAUCCCCAGCGCCAAUGUACAGAUGGGCGCCGGGGAGCAUUUAAUUCAAGCGGGUCCCAUGCAGUGGCGCUUGCAGAGGUCGCAAUCUUCUCGAUU
Rasp1-R2_Rep3 4101 AGGGUAUCCCCAGCGCCAAUGUACAGAUGGGCGCCGGGGAGCAUUUAAUUCAAGCGGGUCCCAUGCAGUGGCGCUUGCAGAGGUCGCAAUCUUCUCGAUU

Rasp1-R2_2014 4201 UGUGGUCUCUGGUCAUUCGCGAACGCGUGGAAGCUCUCUGUUUGCUGGAAGUGUCGAUAGGACGCAGCAGGGAACGGGAGCUUUUGAAGACCCGGGUUUU
Rasp1-R2_Rep1 4201 UGUGGUCUCUGGUCAUUCGCGAACGCGUGGAAGCUCUCUGUUUGCUGGAAGUGUCGAUAGGACGCAGCAGGGAACGGGAGCUUUUGAAGACCCGGGUUUU
Rasp1-R2_Rep2 4201 UGUGGUCUCUGGUCAUUCGCGAACGCGUGGAAGCUCUCUGUUUGCUGGAAGUGUCGAUAGGACGCAGCAGGGAACGGGAGCUUUUGAAGACCCGGGUUUU
Rasp1-R2_Rep3 4201 UGUGGUCUCUGGUCAUUCGCGAACGCGUGGAAGCUCUCUGUUUGCUGGAAGUGUCGAUAGGACGCAGCAGGGAACGGGAGCUUUUGAAGACCCGGGUUUU

Rasp1-R2_2014 4301 UUACCACCCAGGAAUUCUUCUGUUCAGGGCGGAUCCUGGCAAGAAGGUACUGAAGCCGCUUUUUUAGGCAAAGUUACCUGUGCGAAGGACGCCAAGGGUG
Rasp1-R2_Rep1 4301 UUACCACCCAGGAAUUCUUCUGUUCAGGGCGGAUCCUGGCAAGAAGGUACUGAAGCCGCUUUUUUAGGCAAAGUUACCUGUGCGAAGGACGCCAAGGGUG
Rasp1-R2_Rep2 4301 UUACCACCCAGGAAUUCUUCUGUUCAGGGCGGAUCCUGGCAAGAAGGUACUGAAGCCGCUUUUUUAGGCAAAGUUACCUGUGCGAAGGACGCCAAGGGUG
Rasp1-R2_Rep3 4301 UUACCACCCAGGAAUUCUUCUGUUCAGGGCGGAUCCUGGCAAGAAGGUACUGAAGCCGCUUUUUUAGGCAAAGUUACCUGUGCGAAGGACGCCAAGGGUG

Rasp1-R2_2014 4401 GAACUUUAUUGCACACUUUGGAUAUUAUAAAAGAGUGCAAAUCCCAAAAUUUAUUAAGGUAUAAAGAGUGGCAACGUCAAGGCUUUCUUCAUGGAAAGCU
Rasp1-R2_Rep1 4401 GAACUUUAUUGCACACUUUGGAUAUUAUAAAAGAGUGCAAAUCCCAAAAUUUAUUAAGGUAUAAAGAGUGGCAACGUCAAGGCUUUCUUCAUGGAAAGCU
Rasp1-R2_Rep2 4401 GAACUUUAUUGCACACUUUGGAUAUUAUAAAAGAGUGCAAAUCCCAAAAUUUAUUAAGGUAUAAAGAGUGGCAACGUCAAGGCUUUCUUCAUGGAAAGCU
Rasp1-R2_Rep3 4401 GAACUUUAUUGCACACUUUGGAUAUUAUAAAAGAGUGCAAAUCCCAAAAUUUAUUAAGGUAUAAAGAGUGGCAACGUCAAGGCUUUCUUCAUGGAAAGCU

Rasp1-R2_2014 4501 UAGAUUGCGCUGUUUCAUACCCACUAACAUUUUUUGUGGGCAUUCUAUGAUGUGUUCUUUGGAUGCGUUUGGUCGUUAUGAUUCGAGCGUGCUAGGUGCU
Rasp1-R2_Rep1 4501 UAGAUUGCGCUGUUUCAUACCCACUAACAUUUUUUGUGGGCAUUCUAUGAUGUGUUCUUUGGAUGCGUUUGGUCGUUAUGAUUCGAGCGUGCUAGGUGCU
Rasp1-R2_Rep2 4501 UAGAUUGCGCUGUUUCAUACCCACUAACAUUUUUUGUGGGCAUUCUAUGAUGUGUUCUUUGGAUGCGUUUGGUCGUUAUGAUUCGAGCGUGCUAGGUGCU
Rasp1-R2_Rep3 4501 UAGAUUGCGCUGUUUCAUACCCACUAACAUUUUUUGUGGGCAUUCUAUGAUGUGUUCUUUGGAUGCGUUUGGUCGUUAUGAUUCGAGCGUGCUAGGUGCU

Rasp1-R2_2014 4601 AGUUUUCCAGUGAAGUUGGCAAGUUUAUUGCCAACGGAGGUGAUUAGCCUAGCUGAUGGACCCGUGGUCACGUGGACGUUUGAUAUUGGACGUCUGUGUG
Rasp1-R2_Rep1 4601 AGUUUUCCAGUGAAGUUGGCAAGUUUAUUGCCAACGGAGGUGAUUAGCCUAGCUGAUGGACCCGUGGUCACGUGGACGUUUGAUAUUGGACGUCUGUGUG
Rasp1-R2_Rep2 4601 AGUUUUCCAGUGAAGUUGGCAAGUUUAUUGCCAACGGAGGUGAUUAGCCUAGCUGAUGGACCCGUGGUCACGUGGACGUUUGAUAUUGGACGUCUGUGUG
Rasp1-R2_Rep3 4601 AGUUUUCCAGUGAAGUUGGCAAGUUUAUUGCCAACGGAGGUGAUUAGCCUAGCUGAUGGACCCGUGGUCACGUGGACGUUUGAUAUUGGACGUCUGUGUG

Rasp1-R2_2014 4701 GUCAUGGUCUCUAUUAUUCUGAGGGCGCUUAUGCGAGGCCCAAAAUUUACUUUUUAAUUCUUUCCGAUAAUGAUGUUCCUGCAGAAGCAGAUUGGCAAUU
Rasp1-R2_Rep1 4701 GUCAUGGUCUCUAUUAUUCUGAGGGCGCUUAUGCGAGGCCCAAAAUUUACUUUUUAAUUCUUUCCGAUAAUGAUGUUCCUGCAGAAGCAGAUUGGCAAUU
Rasp1-R2_Rep2 4701 GUCAUGGUCUCUAUUAUUCUGAGGGCGCUUAUGCGAGGCCCAAAAUUUACUUUUUAAUUCUUUCCGAUAAUGAUGUUCCUGCAGAAGCAGAUUGGCAAUU
Rasp1-R2_Rep3 4701 GUCAUGGUCUCUAUUAUUCUGAGGGCGCUUAUGCGAGGCCCAAAAUUUACUUUUUAAUUCUUUCCGAUAAUGAUGUUCCUGCAGAAGCAGAUUGGCAAUU

Rasp1-R2_2014 4801 UACCUAUCAGCUUUUGUUUGAGGAUCAUACAUUUUCGAAUUCCUUUGGGGCGGUUCCUUUUAUUACCUUACCCCAUAUUUUUAAUAGAUUAGAUAUAGGU
Rasp1-R2_Rep1 4801 UACCUAUCAGCUUUUGUUUGAGGAUCAUACAUUUUCGAAUUCCUUUGGGGCGGUUCCUUUUAUUACCUUACCCCAUAUUUUUAAUAGAUUAGAUAUAGGU
Rasp1-R2_Rep2 4801 UACCUAUCAGCUUUUGUUUGAGGAUCAUACAUUUUCGAAUUCCUUUGGGGCGGUUCCUUUUAUUACCUUACCCCAUAUUUUUAAUAGAUUAGAUAUAGGU
Rasp1-R2_Rep3 4801 UACCUAUCAGCUUUUGUUUGAGGAUCAUACAUUUUCGAAUUCCUUUGGGGCGGUUCCUUUUAUUACCUUACCCCAUAUUUUUAAUAGAUUAGAUAUAGGU

Rasp1-R2_2014 4901 UAUUGGCGCGGGCCAACAGAGAUAGAUUUAACAUCAACUCCCGCACCGAACGCUUAUCGUUUACUUUUCGGCUUGUCCACUGUUAUUAGUGGUAACAUGU
Rasp1-R2_Rep1 4901 UAUUGGCGCGGGCCAACAGAGAUAGAUUUAACAUCAACUCCCGCACCGAACGCUUAUCGUUUACUUUUCGGCUUGUCCACUGUUAUUAGUGGUAACAUGU
Rasp1-R2_Rep2 4901 UAUUGGCGCGGGCCAACAGAGAUAGAUUUAACAUCAACUCCCGCACCGAACGCUUAUCGUUUACUUUUCGGCUUGUCCACUGUUAUUAGUGGUAACAUGU
Rasp1-R2_Rep3 4901 UAUUGGCGCGGGCCAACAGAGAUAGAUUUAACAUCAACUCCCGCACCGAACGCUUAUCGUUUACUUUUCGGCUUGUCCACUGUUAUUAGUGGUAACAUGU

Rasp1-R2_2014 5001 CGACUUUGAAUGCCAAUCAAGCCCUAUUGCGUUUUUUUCAGGGCUCGAAUGGCACUUUACAUGGGCGCAUUAAAAAGAUAGGGACAGCACUUACAACCUG
Rasp1-R2_Rep1 5001 CGACUUUGAAUGCCAAUCAAGCCCUAUUGCGUUUUUUUCAGGGCUCGAAUGGCACUUUACAUGGGCGCAUUAAAAAGAUAGGGACAGCACUUACAACCUG
Rasp1-R2_Rep2 5001 CGACUUUGAAUGCCAAUCAAGCCCUAUUGCGUUUUUUUCAGGGCUCGAAUGGCACUUUACAUGGGCGCAUUAAAAAGAUAGGGACAGCACUUACAACCUG
Rasp1-R2_Rep3 5001 CGACUUUGAAUGCCAAUCAAGCCCUAUUGCGUUUUUUUCAGGGCUCGAAUGGCACUUUACAUGGGCGCAUUAAAAAGAUAGGGACAGCACUUACAACCUG

Rasp1-R2_2014 5101 UUCCCUUUUACUAUCGUUGCGCCACAAAGAUGCGGAUCUCACAUUGGAGACCGCAUAUCAAAGGCCCCAUUACAUUUUGGCUAACGGACAAGGGGCUUUU
Rasp1-R2_Rep1 5101 UUCCCUUUUACUAUCGUUGCGCCACAAAGAUGCGGAUCUCACAUUGGAGACCGCAUAUCAAAGGCCCCAUUACAUUUUGGCUAACGGACAAGGGGCUUUU
Rasp1-R2_Rep2 5101 UUCCCUUUUACUAUCGUUGCGCCACAAAGAUGCGGAUCUCACAUUGGAGACCGCAUAUCAAAGGCCCCAUUACAUUUUGGCUAACGGACAAGGGGCUUUU
Rasp1-R2_Rep3 5101 UUCCCUUUUACUAUCGUUGCGCCACAAAGAUGCGGAUCUCACAUUGGAGACCGCAUAUCAAAGGCCCCAUUACAUUUUGGCUAACGGACAAGGGGCUUUU

Rasp1-R2_2014 5201 UCAUUACCAAUUUCUACCCCCCAUGCAGCAACCUCCUUUUUAGAGGACAUGUUGCGCCUGGAGAUUUUUGCUAUUGCUGGGCCUUUUAGUCCCAAAGAUA
Rasp1-R2_Rep1 5201 UCAUUACCAAUUUCUACCCCCCAUGCAGCAACCUCCUUUUUAGAGGACAUGUUGCGCCUGGAGAUUUUUGCUAUUGCUGGGCCUUUUAGUCCCAAAGAUA
Rasp1-R2_Rep2 5201 UCAUUACCAAUUUCUACCCCCCAUGCAGCAACCUCCUUUUUAGAGGACAUGUUGCGCCUGGAGAUUUUUGCUAUUGCUGGGCCUUUUAGUCCCAAAGAUA
Rasp1-R2_Rep3 5201 UCAUUACCAAUUUCUACCCCCCAUGCAGCAACCUCCUUUUUAGAGGACAUGUUGCGCCUGGAGAUUUUUGCUAUUGCUGGGCCUUUUAGUCCCAAAGAUA

Rasp1-R2_2014 5301 AUAAUGCAAAAUACCAAUUCAUGUGUUAUUUCGAUCACAUAGAACUGGUUGAGGGGGUACCUAGAACUAUAGCAGGCGAACAGCAGUUCAAUUGGUGUAG
Rasp1-R2_Rep1 5301 AUAAUGCAAAAUACCAAUUCAUGUGUUAUUUCGAUCACAUAGAACUGGUUGAGGGGGUACCUAGAACUAUAGCAGGCGAACAGCAGUUCAAUUGGUGUAG
Rasp1-R2_Rep2 5301 AUAAUGCAAAAUACCAAUUCAUGUGUUAUUUCGAUCACAUAGAACUGGUUGAGGGGGUACCUAGAACUAUAGCAGGCGAACAGCAGUUCAAUUGGUGUAG
Rasp1-R2_Rep3 5301 AUAAUGCAAAAUACCAAUUCAUGUGUUAUUUCGAUCACAUAGAACUGGUUGAGGGGGUACCUAGAACUAUAGCAGGCGAACAGCAGUUCAAUUGGUGUAG

Rasp1-R2_2014 5401 UUUUAGAAAUUUCAAAAUCGAUGACUGGAGGUUUGAGUGGCCGGCUCGCCUUCCAGAUAUACUUGAUGAUAAGUCAGAAGUGCUUUUGAGGCAACAUCCU
Rasp1-R2_Rep1 5401 UUUUAGAAAUUUCAAAAUCGAUGACUGGAGGUUUGAGUGGCCGGCUCGCCUUCCAGAUAUACUUGAUGAUAAGUCAGAAGUGCUUUUGAGGCAACAUCCU
Rasp1-R2_Rep2 5401 UUUUAGAAAUUUCAAAAUCGAUGACUGGAGGUUUGAGUGGCCGGCUCGCCUUCCAGAUAUACUUGAUGAUAAGUCAGAAGUGCUUUUGAGGCAACAUCCU
Rasp1-R2_Rep3 5401 UUUUAGAAAUUUCAAAAUCGAUGACUGGAGGUUUGAGUGGCCGGCUCGCCUUCCAGAUAUACUUGAUGAUAAGUCAGAAGUGCUUUUGAGGCAACAUCCU

Rasp1-R2_2014 5501 UUAUCUCUGCUUAUCUCAUCUACCGGUUUUUUUACGGGUAGAGCCAUUUUCGUUUUCCAGUGGGGUUUGAAUACUACUGCUGGGAAUAUGAAAGGCUCAU
Rasp1-R2_Rep1 5501 UUAUCUCUGCUUAUCUCAUCUACCGGUUUUUUUACGGGUAGAGCCAUUUUCGUUUUCCAGUGGGGUUUGAAUACUACUGCUGGGAAUAUGAAAGGCUCAU
Rasp1-R2_Rep2 5501 UUAUCUCUGCUUAUCUCAUCUACCGGUUUUUUUACGGGUAGAGCCAUUUUCGUUUUCCAGUGGGGUUUGAAUACUACUGCUGGGAAUAUGAAAGGCUCAU
Rasp1-R2_Rep3 5501 UUAUCUCUGCUUAUCUCAUCUACCGGUUUUUUUACGGGUAGAGCCAUUUUCGUUUUCCAGUGGGGUUUGAAUACUACUGCUGGGAAUAUGAAAGGCUCAU

Rasp1-R2_2014 5601 UUUCUGCGCGCCUGGCCUUUGGCAAGGGCGUUGAGGAAAUUGAGCAAACGUCAACAGUGCAACCACUUGUUGGCGCUUGUGAAGCCCGCAUACCCGUGGA
Rasp1-R2_Rep1 5601 UUUCUGCGCGCCUGGCCUUUGGCAAGGGCGUUGAGGAAAUUGAGCAAACGUCAACAGUGCAACCACUUGUUGGCGCUUGUGAAGCCCGCAUACCCGUGGA
Rasp1-R2_Rep2 5601 UUUCUGCGCGCCUGGCCUUUGGCAAGGGCGUUGAGGAAAUUGAGCAAACGUCAACAGUGCAACCACUUGUUGGCGCUUGUGAAGCCCGCAUACCCGUGGA
Rasp1-R2_Rep3 5601 UUUCUGCGCGCCUGGCCUUUGGCAAGGGCGUUGAGGAAAUUGAGCAAACGUCAACAGUGCAACCACUUGUUGGCGCUUGUGAAGCCCGCAUACCCGUGGA

Rasp1-R2_2014 5701 GUUUAAGACUUACACGGGUUAUACUACUUCGGGUCCUCCUGGAUCCAUGGAACCAUACAUUUACGUGAGGCUUACGCAAGCUAAGCUUGUGGAUAGGCUU
Rasp1-R2_Rep1 5701 GUUUAAGACUUACACGGGUUAUACUACUUCGGGUCCUCCUGGAUCCAUGGAACCAUACAUUUACGUGAGGCUUACGCAAGCUAAGCUUGUGGAUAGGCUU
Rasp1-R2_Rep2 5701 GUUUAAGACUUACACGGGUUAUACUACUUCGGGUCCUCCUGGAUCCAUGGAACCAUACAUUUACGUGAGGCUUACGCAAGCUAAGCUUGUGGAUAGGCUU
Rasp1-R2_Rep3 5701 GUUUAAGACUUACACGGGUUAUACUACUUCGGGUCCUCCUGGAUCCAUGGAACCAUACAUUUACGUGAGGCUUACGCAAGCUAAGCUUGUGGAUAGGCUU

Rasp1-R2_2014 5801 UCUGUGAAUGUUAUUUUACAGGAGGGAUUUUCUUUCUAUGGACCUAGCAUCAAACAUUUCAAGAAAGAAGUCGGCACGCCUAGUGCCACCCUAGAGACAA
Rasp1-R2_Rep1 5801 UCUGUGAAUGUUAUUUUACAGGAGGGAUUUUCUUUCUAUGGACCUAGCAUCAAACAUUUCAAGAAAGAAGUCGGCACGCCUAGUGCCACCCUAGAGACAA
Rasp1-R2_Rep2 5801 UCUGUGAAUGUUAUUUUACAGGAGGGAUUUUCUUUCUAUGGACCUAGCAUCAAACAUUUCAAGAAAGAAGUCGGCACGCCUAGUGCCACCCUAGAGACAA
Rasp1-R2_Rep3 5801 UCUGUGAAUGUUAUUUUACAGGAGGGAUUUUCUUUCUAUGGACCUAGCAUCAAACAUUUCAAGAAAGAAGUCGGCACGCCUAGUGCCACCCUAGAGACAA

Rasp1-R2_2014 5901 AUAAUCCCGUUGGGCGCCCACCUGAGAAUGUCGAUACAGGGGGUCCUGGCGGCCAGUAUGCAGCUGCCUUACAAGCAGCUCAGCAAGCUGGGAAAAAUCC
Rasp1-R2_Rep1 5901 AUAAUCCCGUUGGGCGCCCACCUGAGAAUGUCGAUACAGGGGGUCCUGGCGGCCAGUAUGCAGCUGCCUUACAAGCAGCUCAGCAAGCUGGGAAAAAUCC
Rasp1-R2_Rep2 5901 AUAAUCCCGUUGGGCGCCCACCUGAGAAUGUCGAUACAGGGGGUCCUGGCGGCCAGUAUGCAGCUGCCUUACAAGCAGCUCAGCAAGCUGGGAAAAAUCC
Rasp1-R2_Rep3 5901 AUAAUCCCGUUGGGCGCCCACCUGAGAAUGUCGAUACAGGGGGUCCUGGCGGCCAGUAUGCAGCUGCCUUACAAGCAGCUCAGCAAGCUGGGAAAAAUCC

Rasp1-R2_2014 6001 UUUCGGGCGUGGUUAAGUUGGCUUCCUGAGAGGCGAGUAGCUGCCGUUAGCAGCUUCCAAAAGGUGGCCUCUUAAUUAGCUUUUAAUAGGGGUUAUCCAG
Rasp1-R2_Rep1 6001 UUUCGGGCGUGGUUAAGUUGGCUUCCUGAGAGGCGAGUAGCUGCCGUUAGCAGCUUCCAAAAGGUGGCCUCUUAAUUAGCUUUUAAUAGGGGUUAUCCAG
Rasp1-R2_Rep2 6001 UUUCGGGCGUGGUUAAGUUGGCUUCCUGAGAGGCGAGUAGCUGCCGUUAGCAGCUUCCAAAAGGUGGCCUCUUAAUUAGCUUUUAAUAGGGGUUAUCCAG
Rasp1-R2_Rep3 6001 UUUCGGGCGUGGUUAAGUUGGCUUCCUGAGAGGCGAGUAGCUGCCGUUAGCAGCUUCCAAAAGGUGGCCUCUUAAUUAGCUUUUAAUAGGGGUUAUCCAG

Rasp1-R2_2014 6101 CCUUAAGCAAGCUGGCACCGGUCCUGAUGGACUACCAGGAAAGUACCUGGUUUGGAAGAAUUCGAGUAAAAUUCUUAAAUCUUGUUUACUCGUGACUUAU
Rasp1-R2_Rep1 6101 CCUUAAGCAAGCUGGCACCGGUCCUGAUGGACUACCAGGAAAGUACCUGGUUUGGAAGAAUUCGAGUAAAAUUCUUAAAUCUUGUUUACUCGUGACUUAU
Rasp1-R2_Rep2 6101 CCUUAAGCAAGCUGGCACCGGUCCUGAUGGACUACCAGGAAAGUACCUGGUUUGGAAGAAUUCGAGUAAAAUUCUUAAAUCUUGUUUACUCGUGACUUAU
Rasp1-R2_Rep3 6101 CCUUAAGCAAGCUGGCACCGGUCCUGAUGGACUACCAGGAAAGUACCUGGUUUGGAAGAAUUCGAGUAAAAUUCUUAAAUCUUGUUUACUCGUGACUUAU

Rasp1-R2_2014 6201 AGUACAUUCAAGAUGAAUGACUCAUGUUUUGUCCAUUUACAUGAUGGCAUAAAGAGUUAACGGCUCAUAUGGCGCUCAUUACGUUCAAGUGUUGAAGGAU
Rasp1-R2_Rep1 6201 AGUACAUUCAAGAUGAAUGACUCAUGUUUUGUCCAUUUACAUGAUGGCAUAAAGAGUUAACGGCUCAUAUGGCGCUCAUUACGUUCAAGUGUUGAAGGAU
Rasp1-R2_Rep2 6201 AGUACAUUCAAGAUGAAUGACUCAUGUUUUGUCCAUUUACAUGAUGGCAUAAAGAGUUAACGGCUCAUAUGGCGCUCAUUACGUUCAAGUGUUGAAGGAU
Rasp1-R2_Rep3 6201 AGUACAUUCAAGAUGAAUGACUCAUGUUUUGUCCAUUUACAUGAUGGCAUAAAGAGUUAACGGCUCAUAUGGCGCUCAUUACGUUCAAGUGUUGAAGGAU

Rasp1-R2_2014 6301 UCAAUAGCCUUGAACUGUGGUGCCAUGUGAGGAAAUCCACGUUAUCUCUGAUUGUCAAAAUAGACUAGUCUAGGAGACGAUAAAUCUUAUGUGGGUGAGU
Rasp1-R2_Rep1 6301 UCAAUAGCCUUGAACUGUGGUGCCAUGUGAGGAAAUCCACGUUAUCUCUGAUUGUCAAAAUAGACUAGUCUAGGAGACGAUAAAUCUUAUGUGGGUGAGU
Rasp1-R2_Rep2 6301 UCAAUAGCCUUGAACUGUGGUGCCAUGUGAGGAAAUCCACGUUAUCUCUGAUUGUCAAAAUAGACUAGUCUAGGAGACGAUAAAUCUUAUGUGGGUGAGU
Rasp1-R2_Rep3 6301 UCAAUAGCCUUGAACUGUGGUGCCAUGUGAGGAAAUCCACGUUAUCUCUGAUUGUCAAAAUAGACUAGUCUAGGAGACGAUAAAUCUUAUGUGGGUGAGU

Rasp1-R2_2014 6401 CCCAUUCUGGCGAGAUACGCAAUGCCUUUUAUUUGUUUGAGGUUAUCAAACAUCAUAUCUUGAGUCUGCGUUUAAAUUUCAAUAAUGUAGUUGUCAUAGC
Rasp1-R2_Rep1 6401 CCCAUUCUGGCGAGAUACGCAAUGCCUUUUAUUUGUUUGAGGUUAUCAAACAUCAUAUCUUGAGUCUGCGUUUAAAUUUCAAUAAUGUAGUUGUCAUAGC
Rasp1-R2_Rep2 6401 CCCAUUCUGGCGAGAUACGCAAUGCCUUUUAUUUGUUUGAGGUUAUCAAACAUCAUAUCUUGAGUCUGCGUUUAAAUUUCAAUAAUGUAGUUGUCAUAGC
Rasp1-R2_Rep3 6401 CCCAUUCUGGCGAGAUACGCAAUGCCUUUUAUUUGUUUGAGGUUAUCAAACAUCAUAUCUUGAGUCUGCGUUUAAAUUUCAAUAAUGUAGUUGUCAUAGC

Rasp1-R2_2014 6501 CUACCGAUGAACCUGCGAGAAAGGUUCCAUGAGGACUAGGGUUGGCUAACCCUCACUUAAUCUCUCUAUUGGUCAUUCGACAGUGUGUCGAGAAUUCAUG
Rasp1-R2_Rep1 6501 CUACCGAUGAACCUGCGAGAAAGGUUCCAUGAGGACUAGGGUUGGCUAACCCUCACUUAAUCUCUCUAUUGGUCAUUCGACAGUGUGUCGAGAAUUCAUG
Rasp1-R2_Rep2 6501 CUACCGAUGAACCUGCGAGAAAGGUUCCAUGAGGACUAGGGUUGGCUAACCCUCACUUAAUCUCUCUAUUGGUCAUUCGACAGUGUGUCGAGAAUUCAUG
Rasp1-R2_Rep3 6501 CUACCGAUGAACCUGCGAGAAAGGUUCCAUGAGGACUAGGGUUGGCUAACCCUCACUUAAUCUCUCUAUUGGUCAUUCGACAGUGUGUCGAGAAUUCAUG

Rasp1-R2_2014 6601 GGUUUCAUCACCCACAUUGAAGCGAGUGUCUCGUAAGAAACCACUCGGAUUGGUGUACUUACCAUGCAUCCUUUCGAGUAAAGCAUCGAUUCGUCGUUGU
Rasp1-R2_Rep1 6601 GGUUUCAUCACCCACAUUGAAGCGAGUGUCUCGUAAGAAACCACUCGGAUUGGUGUACUUACCAUGCAUCCUUUCGAGUAAAGCAUCGAUUCGUCGUUGU
Rasp1-R2_Rep2 6601 GGUUUCAUCACCCACAUUGAAGCGAGUGUCUCGUAAGAAACCACUCGGAUUGGUGUACUUACCAUGCAUCCUUUCGAGUAAAGCAUCGAUUCGUCGUUGU
Rasp1-R2_Rep3 6601 GGUUUCAUCACCCACAUUGAAGCGAGUGUCUCGUAAGAAACCACUCGGAUUGGUGUACUUACCAUGCAUCCUUUCGAGUAAAGCAUCGAUUCGUCGUUGU

Rasp1-R2_2014 6701 GGUUCUUCAAUUGUGGUUUUAGAUGAGCGAUGAGAUCGCUGGCCGCGUUAGAGCGUGAAAAGUAGUCUGAAACGAACUUAGUACCAGAGGUAGGACGCCA
Rasp1-R2_Rep1 6701 GGUUCUUCAAUUGUGGUUUUAGAUGAGCGAUGAGAUCGCUGGCCGCGUUAGAGCGUGAAAAGUAGUCUGAAACGAACUUAGUACCAGAGGUAGGACGCCA
Rasp1-R2_Rep2 6701 GGUUCUUCAAUUGUGGUUUUAGAUGAGCGAUGAGAUCGCUGGCCGCGUUAGAGCGUGAAAAGUAGUCUGAAACGAACUUAGUACCAGAGGUAGGACGCCA
Rasp1-R2_Rep3 6701 GGUUCUUCAAUUGUGGUUUUAGAUGAGCGAUGAGAUCGCUGGCCGCGUUAGAGCGUGAAAAGUAGUCUGAAACGAACUUAGUACCAGAGGUAGGACGCCA

Rasp1-R2_2014 6801 UUGUUCCAGGCGUUUUUUAUGGGCAUAAGCUGUAAAUUUGGUUUCGCAAGCCAUUCAGCACCUCCCUUUAUUCGUGUACUAUCCAGGGGCUCCCGGUUCU
Rasp1-R2_Rep1 6801 UUGUUCCAGGCGUUUUUUAUGGGCAUAAGCUGUAAAUUUGGUUUCGCAAGCCAUUCAGCACCUCCCUUUAUUCGUGUACUAUCCAGGGGCUCCCGGUUCU
Rasp1-R2_Rep2 6801 UUGUUCCAGGCGUUUUUUAUGGGCAUAAGCUGUAAAUUUGGUUUCGCAAGCCAUUCAGCACCUCCCUUUAUUCGUGUACUAUCCAGGGGCUCCCGGUUCU
Rasp1-R2_Rep3 6801 UUGUUCCAGGCGUUUUUUAUGGGCAUAAGCUGUAAAUUUGGUUUCGCAAGCCAUUCAGCACCUCCCUUUAUUCGUGUACUAUCCAGGGGCUCCCGGUUCU

Rasp1-R2_2014 6901 UUCUUACCGGUACAAUACCUGGCGAAGCGAAUAUUGCGUCGAGGGAUGAGAGUAGCAUGUUCCUGCUCACUGAAGGAAUAUGUCGUGUUUUCUACACGUU
Rasp1-R2_Rep1 6901 UUCUUACCGGUACAAUACCUGGCGAAGCGAAUAUUGCGUCGAGGGAUGAGAGUAGCAUGUUCCUGCUCACUGAAGGAAUAUGUCGUGUUUUCUACACGUU
Rasp1-R2_Rep2 6901 UUCUUACCGGUACAAUACCUGGCGAAGCGAAUAUUGCGUCGAGGGAUGAGAGUAGCAUGUUCCUGCUCACUGAAGGAAUAUGUCGUGUUUUCUACACGUU
Rasp1-R2_Rep3 6901 UUCUUACCGGUACAAUACCUGGCGAAGCGAAUAUUGCGUCGAGGGAUGAGAGUAGCAUGUUCCUGCUCACUGAAGGAAUAUGUCGUGUUUUCUACACGUU

Rasp1-R2_2014 7001 AGUGUUAUGACGCUACCCAGCGCCAUAGUGCAAGAAUGGUUCCCAGCCACUUUUUCUGGGAUUCUAAUCGUACGACACAAUUGCAUGUGUACUGUUGACG
Rasp1-R2_Rep1 7001 AGUGUUAUGACGCUACCCAGCGCCAUAGUGCAAGAAUGGUUCCCAGCCACUUUUUCUGGGAUUCUAAUCGUACGACACAAUUGCAUGUGUACUGUUGACG
Rasp1-R2_Rep2 7001 AGUGUUAUGACGCUACCCAGCGCCAUAGUGCAAGAAUGGUUCCCAGCCACUUUUUCUGGGAUUCUAAUCGUACGACACAAUUGCAUGUGUACUGUUGACG
Rasp1-R2_Rep3 7001 AGUGUUAUGACGCUACCCAGCGCCAUAGUGCAAGAAUGGUUCCCAGCCACUUUUUCUGGGAUUCUAAUCGUACGACACAAUUGCAUGUGUACUGUUGACG

Rasp1-R2_2014 7101 GAGGAGUAGCGAUCCUCUAUCACGCAGGGCCGGAAGUAAUUCCCGGGGCCGAAGAAGGCCAGCAUGCGGUACGAUUAACUUUAGCUGUAAUGUAGUGGUA
Rasp1-R2_Rep1 7101 GAGGAGUAGCGAUCCUCUAUCACGCAGGGCCGGAAGUAAUUCCCGGGGCCGAAGAAGGCCAGCAUGCGGUACGAUUAACUUUAGCUGUAAUGUAGUGGUA
Rasp1-R2_Rep2 7101 GAGGAGUAGCGAUCCUCUAUCACGCAGGGCCGGAAGUAAUUCCCGGGGCCGAAGAAGGCCAGCAUGCGGUACGAUUAACUUUAGCUGUAAUGUAGUGGUA
Rasp1-R2_Rep3 7101 GAGGAGUAGCGAUCCUCUAUCACGCAGGGCCGGAAGUAAUUCCCGGGGCCGAAGAAGGCCAGCAUGCGGUACGAUUAACUUUAGCUGUAAUGUAGUGGUA

Rasp1-R2_2014 7201 UGUUAAGUUGAGACUAACUUACCCGUACGAGUCAAACUUUAAGAUGGAUGUGUGUUCUGCCAUCUUGAGGGAAGUAGGUGUGGUUUUACCAAUCUGAGAC
Rasp1-R2_Rep1 7201 UGUUAAGUUGAGACUAACUUACCCGUACGAGUCAAACUUUAAGAUGGAUGUGUGUUCUGCCAUCUUGAGGGAAGUAGGUGUGGUUUUACCAAUCUGAGAC
Rasp1-R2_Rep2 7201 UGUUAAGUUGAGACUAACUUACCCGUACGAGUCAAACUUUAAGAUGGAUGUGUGUUCUGCCAUCUUGAGGGAAGUAGGUGUGGUUUUACCAAUCUGAGAC
Rasp1-R2_Rep3 7201 UGUUAAGUUGAGACUAACUUACCCGUACGAGUCAAACUUUAAGAUGGAUGUGUGUUCUGCCAUCUUGAGGGAAGUAGGUGUGGUUUUACCAAUCUGAGAC

Rasp1-R2_2014 7301 GAGCCGUUAAUUCGGUGCUUUAAUACGUCAAUGAUAAUACUCGUGCAGUUGCAGCUGCACGAGUAUGUUGGUACACACAGUCUACUCGGAUACGGUCGAG
Rasp1-R2_Rep1 7301 GAGCCGUUAAUUCGGUGCUUUAAUACGUCAAUGAUAAUACUCGUGCAGUUGCAGCUGCACGAGUAUGUUGGUACACACAGUCUACUCGGAUACGGUCGAG
Rasp1-R2_Rep2 7301 GAGCCGUUAAUUCGGUGCUUUAAUACGUCAAUGAUAAUACUCGUGCAGUUGCAGCUGCACGAGUAUGUUGGUACACACAGUCUACUCGGAUACGGUCGAG
Rasp1-R2_Rep3 7301 GAGCCGUUAAUUCGGUGCUUUAAUACGUCAAUGAUAAUACUCGUGCAGUUGCAGCUGCACGAGUAUGUUGGUACACACAGUCUACUCGGAUACGGUCGAG

Rasp1-R2_2014 7401 UUGCCCUCACAACAGGGAUUACUCUCUCAAUCUUAACUACUGCAAGGACGUUGUUUUCGCAGGGUUUUGUUGGUCCGUUUGUGUUUCAAAACGCUGCUUU
Rasp1-R2_Rep1 7401 UUGCCCUCACAACAGGGAUUACUCUCUCAAUCUUAACUACUGCAAGGACGUUGUUUUCGCAGGGUUUUGUUGGUCCGUUUGUGUUUCAAAACGCUGCUUU
Rasp1-R2_Rep2 7401 UUGCCCUCACAACAGGGAUUACUCUCUCAAUCUUAACUACUGCAAGGACGUUGUUUUCGCAGGGUUUUGUUGGUCCGUUUGUGUUUCAAAACGCUGCUUU
Rasp1-R2_Rep3 7401 UUGCCCUCACAACAGGGAUUACUCUCUCAAUCUUAACUACUGCAAGGACGUUGUUUUCGCAGGGUUUUGUUGGUCCGUUUGUGUUUCAAAACGCUGCUUU

Rasp1-R2_2014 7501 GCAAUUUUCUUUUUUUGUUUUAUUGCUUUCGUAGUGUCGAACUUUGUCCAAGUUCAUAAAAGC
Rasp1-R2_Rep1 7501 GCAAUUUUCUUUUUUUGUUUUAUUGCUUUCGUAGUGUCGAACUUUGUCCAAGUUCAUAAAAGC
Rasp1-R2_Rep2 7501 GCAAUUUUCUUUUUUUGUUUUAUUGCUUUCGUAGUGUCGAACUUUGUCCAAGUUCAUAAAAGC
Rasp1-R2_Rep3 7501 GCAAUUUUCUUUUUUUGUUUUAUUGCUUUCGUAGUGUCGAACUUUGUCCAAGUUCAUAAAAGC
