# Supplementary material for: Transcriptomic changes associated with infection of Nicotiana benthamiana plants with tomato ringspot virus (genus Nepovirus) during the acute symptomatic stage and after symptom recovery
Source: PLoS One. 2025 Sep 2;20(9):e0328517. doi: 10.1371/journal.pone.0328517 (PMC12404439; doi:10.1371/journal.pone.0328517)
Supplement: S1 Table — (DOCX) [file pone.0328517.s012.docx]

**S1 Table. List of primers**

|  |  | Primer | Annealing temperature (^o^ C) | Target DEGs |
| --- | --- | --- | --- | --- |
| 1 | HSP70_1903F | GATTGGTTGGACGGGAAC | 52 | Heat shock protein 70kDa (Niben101Scf02124g01003) |
|  | HSP70_2062R | CATAACCAGCTCCAGTGTCA |  |  |
| 2 | PPc-t_1582F | CTGTGTACCAAGGTGTTGGA | 52 | Peptidyl-prolyl cis-trans isomerase D (Niben101Scf01832g01020) |
|  | PPc-t_1691R | TTAATGTCAAATTCAGCCAGATC |  |  |
| 3 | SAR8_151F | CTCCAACTAGCTGATGCAAG | 52 | mRNA inducible by salicylic acid (Niben101Scf06525g03013) |
|  | SAR8_282R | GACACAACATCTGCAAGC |  |  |
| 4 | MORN_1144F | GCAATATAGGCAAGGATTGAGAC | 60 | MORN-motif repeat protein similar to AT1G21920.1 MORN3 or MRF1 protein (Niben101Scf12277g02009) |
|  | MORN_1265R | CATCAGAGCAAGACTGAGACC |  |  |
| 5 | BIM2_764F | AACACATATGCCGCTTCAG | 53 | Transcription factor AP-4 (Niben101Scf08394g00001) |
|  | BIM2_891R | CCTCGTGATGATTCAGAGC |  |  |
| 6 | SP1_955F | AAGGTGTTGAACGTTTGTTCT | 53 | Signal peptidase I (Niben101Scf01487g04011) |
|  | SP1_1083R | ATCGAGAGTAGGTGACATGG |  |  |
| 7 | TIM_561F | ATACAATCTTGAATCAATTCCAGG | 52 | Mitochondrial import inner membrane translocase subunit Tim17/Tim22/Tim23 family protein (Niben101Scf27837g00005) |
|  | TIM_749R | ACTTTATGCCATGTATCAATTATGT |  |  |
| 8 | FREE_137F | CAAGGCGATTACACCAGTTC | 52 | Zinc finger FYVE domain-containing protein 21 (Niben101Scf09812g01018) |
|  | FREE_284R | AAGAGGGAGTGTAAGTGGC |  |  |
| 9 | ARP_323F | GGATTACCAGGGACAGAAGATG | 52 | Auxin-responsive protein IAA4 (Niben101Scf00286g01008) |
|  | ARP_445R | CCATCCCACTATTTGTGTCTTC |  |  |
| 10 | AMD_473F | TACTACTGCTGGATCTTATGCT | 54 | Amidotransferase similar to bacterial Glutamyl-tRNA (Gln) amidotransferase subunit A (Niben101Scf01847g05004) |
|  | AMD_633R | CACACCAACCATTAGGAACTC |  |  |
| 11 | DFL_266 | TTGTGAATGATTGTAGATGTGC | 52 | Defensin-like (Niben101Scf06275g03010) |
|  | DFL_411R | CTTCTGAGGATTTTGCTACAATGA |  |  |
| 12 | ToRSV1_6209F | GATTGTTGCTGATAGACTCGC | 52 | RNA1 (ToRSV) |
|  | ToRSV1_6362R | CGTAGCTTGTTGTCCTGTAC |  |  |
| 13 | ToRSV2_5578F | TGCTGGGAATATGAAAGGC | 52 | RNA2 (ToRSV) |
|  | ToRSV2_5758R | GTATGGTTCCATGGATCCAG |  |  |
| 14 | LdAct_F1 | TCCTGATGGGCAAGTGATTAC | 55 | Actin : primers from Liu et al. (2012) |
|  | LdAct_R1 | TTGTATGTGGTCTCGTGGATTC |  |  |
| 15 | LdPP2A_F1 | GACCCTGATGTTGATGTTCGCT | 52 | Protein phosphatase 2A : primers from Liu et al. (2012) |
|  | LdPP2A_R1 | GAGGGATTTGAAGAGAGATTTC |  |  |

Liu, D., Shi, L., Han, C., Yu, J., Li, D., Zhang, Y., 2012. Validation of reference genes for gene expression studies in virus-infected Nicotiana benthamiana using quantitative real-time PCR. PLoS One 7, e46451.
