# Supplementary material for: Transcriptomic changes associated with infection of Nicotiana benthamiana plants with tomato ringspot virus (genus Nepovirus) during the acute symptomatic stage and after symptom recovery
Source: PLoS One. 2025 Sep 2;20(9):e0328517. doi: 10.1371/journal.pone.0328517 (PMC12404439; doi:10.1371/journal.pone.0328517)
Supplement: Attachment — Submitted filename: Review of Manuscript ToSWV.docx [file pone.0328517.s013.docx]

**Review of Manuscript**

Transcriptomic changes associated with infection of *Nicotiana benthamiana* plants with Tomato Ringspot Virus (genus *Nepovirus*) during the acute symptomatic stage and after symptom recovery

**Summary**

This study investigates transcriptomic changes in *Nicotiana benthamiana* during acute infection and symptom recovery stages of Tomato Ringspot Virus (ToRSV) infection. The authors have identified extensive transcriptome reprogramming during the symptomatic phase, with stress-response gene upregulation and chloroplast-related gene downregulation. Recovery involves a return to baseline for most genes but highlights sustained or induced expression of defensin-like genes and specific miRNAs. The work provides insights into molecular mechanisms underlying symptom dynamics and recovery.

**Strengths**

1. **Novelty:** The focus on transcriptomic changes during symptom recovery adds valuable data to a less-studied phase of plant-virus interactions. Identification of defensin-like genes and miRNAs (e.g., miR391, miR1919) specifically upregulated during recovery offers new candidates for mechanistic studies.
2. **Methodology:** Multi-replicate RNA-seq and small RNA-seq with validation via ddPCR has strengthen the findings. Integration of viral RNA dynamics, vsiRNA hotspots, and host transcriptome has provided a comprehensive view.
3. **Contextualization:** Comparisons with prior studies (e.g., GFLV, CMV) highlight conserved and unique aspects of nepovirus infections. Linking miRNA expression to predicted targets (e.g., miR530 and stress-associated proteins) bridges transcriptomic changes to functional hypotheses.
4. **Data Accessibility:** The reproducibility is enhanced by public deposition of sequencing data (NCBI BioProject PRJNA1253310)

**Weaknesses and Concerns**

1. **Replicate Variability:** The third biological replicate (2018) showed divergent sequencing depth and PCA clustering. While environmental factors are proposed as a cause, additional validation (e.g., more replicates or controlled growth conditions) would strengthen confidence in the conclusions.
2. **miRNA Target Validation:** Predicted miRNA targets (e.g., miR530 and zinc finger A20/AN1 protein) lack experimental validation (e.g., RACE assays or silencing experiments). This limits mechanistic interpretations.
3. **Functional Insights:** The role of defensins/thionins in symptom recovery is highlighted but not experimentally tested. Overexpression or knockout experiments could clarify their contribution to recovery.
4. **Environmental Controls:** Temperature is noted to influence symptom severity, but the study does not explore how transcriptomic changes are directly temperature-dependent. A temperature-controlled comparison (e.g., 21°C vs. 27°C) would add depth.

**Discussion Depth:**

The discussion could better integrate how sustained defensin expression or miRNA-mediated regulation contributes to recovery. For example, do defensins directly inhibit viral replication or modulate plant immunity?

**Recommendations**

1. **Address Replicate Variability:** Include a supplementary analysis (e.g., hierarchical clustering or batch effect correction) to confirm that biological conclusions are robust despite technical differences between replicates.
2. **Validate Key Findings:** Perform functional assays (e.g., VIGS or CRISPR) to test the role of defensins or miR530 targets in symptom recovery. Validate miRNA-target interactions experimentally (e.g., dual-luciferase assays).
3. **Expand Discussion:** Elaborate on the potential mechanisms by which defensins or miRNAs mediate recovery (e.g., antimicrobial activity, signaling cross-talk). Discuss how chloroplast suppression during symptoms might synergize with defense responses.
4. **Clarify Methods:** Specify how "equivalent leaves" were selected during sampling to ensure consistency. Provide more details on small RNA library preparation (e.g., adapter sequences, size selection).

**Ethics and Compliance**

No ethical concerns. Competing interests and funding sources are appropriately declared. Data availability complies with journal policies.

**Conclusion**

This manuscript presents a well-executed transcriptomic analysis of ToRSV-infected *N. benthamiana*, offering novel insights into symptom recovery. While the findings are compelling, additional functional validation and deeper mechanistic exploration would elevate the impact. With revisions addressing the above points, this work will be a valuable contribution to plant virology.

**Recommendation: Minor Revisions**
